# Healthy dietary patterns for prevention of neuropsychiatric disorders: role of inflammatory and metabolic mechanisms

**DOI:** 10.1038/s41538-025-00623-4

**Published:** 2025-12-09

**Authors:** Yinghao Xu, Ziye Ren, Huimin Cai, Xiaofeng Fu, Shuiyue Quan, Weiyun Zhang, Yumei Geng, Qing Tian, Chengyu An, Jiaqi Li, Longfei Jia

**Affiliations:** Department of Neurology & Innovation Center for Neurological Disorders, Xuanwu Hospital, Capital Medical University, National Clinical Research Center for Geriatric Diseases, Beijing, China

**Keywords:** Diseases, Health care, Neurology, Neuroscience, Risk factors

## Abstract

Diet is increasingly being recognized as a modifiable lifestyle factor that plays an important role in neuropsychiatric health. However, existing research has focused on single foods or dietary patterns in relation to specific diseases, limiting their ability to inform precise dietary recommendations. Based on UK Biobank data, we applied Cox regression to assess associations between four healthy dietary patterns and risks of dementia, depression, and anxiety. Moreover, we utilized structural equation modeling to explore potential biological pathways. Our results indicated that Alternate Mediterranean Diet (AMED) demonstrated the strongest protective effect against all-cause dementia (0.92 (0.85–0.99)). Regarding depression, the AMED (0.93 (0.89–0.98)) and Alternative Healthy Eating Index-2010 (AHEI-2010) (0.93 (0.90–0.97)) showed the strongest negative correlation. AMED was also most strongly associated with reduced risk of phobic anxiety disorders (0.87 (0.77–0.99)), whereas AHEI-2010 exhibited the most pronounced inverse association with other anxiety disorders (0.94 (0.91–0.98)). Structural equation modeling indicated that healthy dietary patterns may reduce the risk of neuropsychiatric disorders partly through direct modulation of inflammation and partly by improving metabolic function, which in turn reduces inflammation. Collectively, our findings support the development of precision-targeted dietary recommendations for the diet-immune-brain axis.

## Introduction

The prevalence of dementia has been increasing with the aging population, causing a large burden on health and social care^1,2^. Depression and anxiety are prevalent neuropsychiatric disorders that affect mental health, social functioning, and overall quality of life^3^. Diet is a modifiable and cost-effective lifestyle factor, with proven effects on brain health^4–6^. Numerous studies have shown significant associations between various healthy dietary patterns and reduced risk of dementia, depression, and anxiety^7,8^. However, because many observational studies and clinical trials have focused on specific foods or dietary patterns, comprehensively evaluating the effects of various dietary patterns across multiple diseases and precisely identifying the optimal intervention for a given condition remain challenging. Therefore, a unified analytical framework is required to evaluate multiple dietary patterns in relation to a range of neuropsychiatric disorders.

Previous studies have shown that a healthy diet is associated with reduced inflammatory responses^9–11^. Higher adherence to Alternate Mediterranean Diet (AMED) and Healthy Eating Index 2015 (HEI-2015) might reduce risk of Crohn’s disease (CD) partly by their anti-inflammatory properties^12^. Evidence also indicates that a diet characterized by high-quality plant-based foods and lower intakes of animal products might be associated with lower risks of total mortality, cancer, and cardiovascular disease (CVD), which may partly be attributed to the anti-inflammatory effects of plant-derived foods^13^. Additionally, specific nutrients, such as antioxidants (e.g., vitamins C and E) and omega-3 fatty acids, have been shown to reduce chronic inflammation, whereas diets rich in sugars and processed foods may aggravate inflammatory responses^14^. Inflammatory responses are implicated in the pathophysiology of several neuropsychiatric disorders^15,16^. Furthermore, metabolic processes are likely to serve as key biological pathways through which diet influences nervous system functions^17^. However, the associations between multiple dietary patterns and disease outcomes have only been systematically evaluated in a few studies. Moreover, the mediating role of inflammation in each specific diet-disease relationship has been quantified and compared in only a few studies.

Therefore, we aimed to utilize data from the UK Biobank (UKB)—a large-scale prospective cohort with detailed assessments, including lifestyle questionnaires and biochemical markers—to investigate the impact of diet on brain health^18^. Specifically, we included four well-established and widely recommended dietary patterns: the AMED, Alternative Healthy Eating Index-2010 (AHEI-2010), Dietary Approaches to Stop Hypertension (DASH), and healthful Plant-based Diet Index (hPDI). This study has two main objectives. First, we systematically examined the association between these four dietary patterns and the risk of dementia, depression, and anxiety. Second, we explored the mediating roles of inflammation and metabolic processes by conducting parallel mediation analyses to quantify and compare their contributions to each diet-disease relationship. By integrating dietary, clinical, and biomarker data under a unified biological framework, we sought to identify the most effective dietary strategies for disease prevention and illuminate the shared inflammatory pathways linking diet to brain health.

## Results

### Study participants

Participants without complete baseline demographic data (*n* = 53,291) or dietary questionnaire responses (*n* = 291,359) were excluded. Specifically, individuals with extreme energy intake (*n* = 1755; definition: <500 or >3500 kcal/day for women and <800 or >4200 kcal/day for men) were excluded from the analysis (Supplementary Figure 1)^19,20^. The baseline characteristics of the study population, categorized into the lowest and highest third percentiles of each dietary pattern score, are presented in Table 1. Participants with high healthy dietary pattern scores were generally more educated, less likely to be current smokers, and had lower BMI than those with low healthy dietary pattern scores. Furthermore, participants in the highest third percentile of hPDI tended to have low total energy intake. Participants with high AMED or AHEI-2010 scores were likely to be female.

**Table 1:** Characteristics of the participants from the UK Biobank study

	AHEI-2010		AMED		DASH		hPDI	
	Quintile1	Quintile3	Quintile1	Quintile3	Quintile1	Quintile3	Quintile1	Quintile3
Sex (Female)	20,352 (43.8)	29,415 (63.3)	27,961 (50.8)	13,332 (60.4)	28,123 (53.6)	23,170 (53.5)	18,299 (51.5)	14,640 (55.4)
Age	54.33 (8.07)	56.52 (7.65)	55.07 (7.94)	56.84 (7.60)	53.97 (8.01)	57.03 (7.55)	54.93 (8.04)	57.20 (7.39)
Smoking status								
Current	4670 (10.1)	2470 (5.3)	5016 (9.1)	949 (4.3)	5423 (10.3)	2067 (4.8)	2801 (7.9)	1597 (6.0)
Former	15,864 (34.1)	16,725 (36.0)	18,821 (34.2)	7878 (35.7)	18,150 (34.6)	15,414 (35.6)	11,757 (33.1)	9779 (37.0)
Never	25,932 (55.8)	27,264 (58.7)	31,207 (56.7)	13,239 (60.0)	28,931 (55.1)	25,856 (59.7)	20983 (59.0)	15073 (57.0)
Alcohol status								
Current	43,199 (93.0)	44,468 (95.7)	52,102 (94.7)	20,723 (93.9)	49,875 (95.0)	40,639 (93.8)	33,574 (94.5)	24,860 (94.0)
Fromer	1631 (3.5)	1042 (2.2)	1473 (2.7)	709 (3.2)	1292 (2.5)	1371 (3.2)	935 (2.6)	846 (3.2)
Never	1636 (3.5)	949 (2.0)	1469 (2.7)	634 (2.9)	1337 (2.5)	1327 (3.1)	1032 (2.9)	743 (2.8)
Education								
High	20,919 (45.0)	24,882 (53.6)	25,500 (46.3)	12,484 (56.6)	246,35 (46.9)	22552 (52.0)	16599 (46.7)	13701 (51.8)
Intermediate	5285 (11.4)	4754 (10.2)	6193 (11.3)	2202 (10.0)	5559 (10.6)	4801 (11.1)	3805 (10.7)	3058 (11.6)
Low	20,262 (43.6)	16,823 (36.2)	23,351 (42.4)	7380 (33.4)	22,310 (42.5)	15,984 (36.9)	15,137 (42.6)	9690 (36.6)
BMI	27.57 (4.79)	25.84 (4.15)	27.15 (4.66)	25.65 (4.09)	27.38 (4.88)	26.02 (4.13)	27.10 (4.73)	26.29 (4.32)
Ethnic group								
White	42,396 (91.2)	41,957 (90.3)	50,206 (91.2)	19,855 (90.0)	47,401 (90.3)	39,662 (91.5)	32,593 (91.7)	24,066 (91.0)
Asian	2009 (4.3)	2560 (5.5)	2500 (4.5)	1265 (5.7)	2733 (5.2)	1978 (4.6)	1583 (4.5)	1203 (4.5)
Other ethnicities	2061 (4.4)	1942 (4.2)	2338 (4.2)	946 (4.3)	2370 (4.5)	1697 (3.9)	1365 (3.8)	1180 (4.5)
Deprivation index								
1	11,344 (24.4)	11,747 (25.3)	13,700 (24.9)	5616 (25.5)	12,370 (23.6)	11,504 (26.5)	8894 (25.0)	6705 (25.4)
2	11,433 (24.6)	11,614 (25.0)	13,652 (24.8)	5389 (24.4)	12,641 (24.1)	11,130 (25.7)	8893 (25.0)	6847 (25.9)
3	11,626 (25.0)	11,739 (25.3)	13,907 (25.3)	5559 (25.2)	13,190 (25.1)	10,822 (25.0)	9087 (25.6)	6644 (25.1)
4	12,063 (26.0)	11,359 (24.4)	13,784 (25.0)	5502 (24.9)	14,302 (27.2)	9881 (22.8)	8666 (24.4)	6253 (23.6)
Sleep time	7.15 (1.00)	7.18 (0.95)	7.17 (0.99)	7.18 (0.94)	7.15 (1.00)	7.18 (0.95)	7.18 (0.98)	7.16 (0.97)
Energy	2152.35 (573.18)	2001.54 (507.29)	2004.04 (541.84)	2125.62 (511.65)	2124.84 (566.85)	2019.53 (507.73)	2203.53 (531.68)	1891.96 (482.21)
Physical activity	2281.18 (2335.14)	2610.07 (2380.06)	2284.43 (2303.74)	2721.03 (2402.17)	2259.69 (2304.63)	2651.39 (2412.08)	2254.53 (2295.35)	2693.45 (2452.55)
APOE 4	0.28 (0.45)	0.29 (0.45)	0.28 (0.45)	0.29 (0.45)	0.28 (0.45)	0.29 (0.45)	0.28 (0.45)	0.28 (0.45)

### Dietary patterns and major neuropsychiatric disorders

**Fig. 1:**
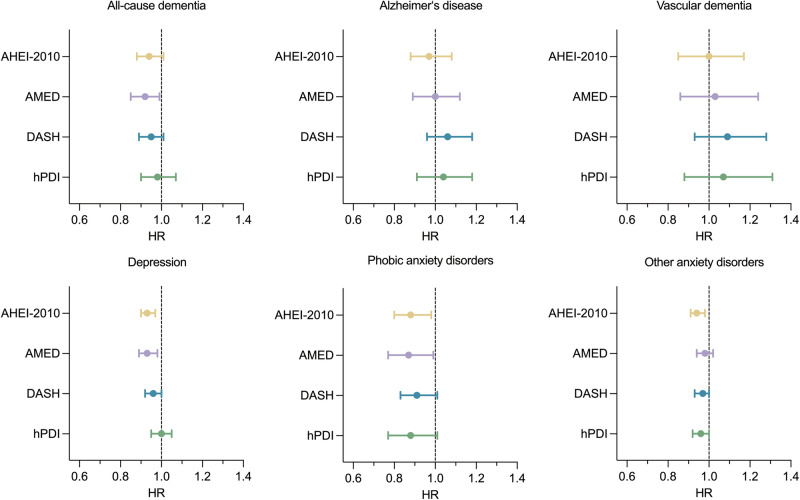
Associations of cumulative average dietary patterns (comparing the highest tertile with the lowest tertile) with major neuropsychiatric disorders and secondary outcomes in the cohort, represented as hazard ratios with 95% confidence intervals. Models were adjusted for age, sex, smoking status, alcohol consumption status, ethnicity, APOEε4, energy intake, education, sleep duration, physical activity, body mass index, and Townsend deprivation index. The analysis details and corresponding estimates are provided in Supplementary Tables 6–11. AMED Alternate Mediterranean Diet, AHEI-2010 Alternative Healthy Eating Index-2010, DASH Dietary Approaches to Stop Hypertension, hPDI healthful Plant-based Diet Index, HR hazard ratio.

We investigated the association between dietary pattern scores and each component of the major neuropsychiatric disorders (Fig. 1). In general, among the various outcomes (dementia, depression, phobic anxiety disorders, and other anxiety disorders), the strongest relationship was between dietary patterns and phobic anxiety disorders. Participants with high adherence to AMED exhibited a lower risk of all-cause dementia (0.92 (0.85–0.99)). However, when analyzing individual types of dementia, although different dietary patterns showed certain trends, no significant association was observed between these dietary patterns and the risk of Alzheimer’s disease (AD) or vascular dementia (VaD) after adjustment for age, sex, smoking status, alcohol consumption, ethnicity, total energy intake, education, sleep duration, physical activity (PA), body mass index (BMI), APOEε4 genotype, and Townsend deprivation index. Increased adherence to AHEI-2010, AMED, or DASH was associated with a reduced risk of depression (AHEI-2010: 0.93 (0.90–0.97), AMED: 0.93 (0.89–0.98), DASH: 0.96 (0.92–1.00)). Participants with high AHEI-2010 and AMED scores exhibited a low risk of developing phobic anxiety disorders (AHEI-2010: 0.88 (0.80–0.98), AMED: 0.87 (0.77–0.99)). Moreover, increased adherence to AHEI-2010 was inversely associated with the risk of other anxiety disorders (0.94 (0.91–0.98)). The results remained largely unchanged after further adjustment for baseline medical history of cardiovascular and cerebrovascular disease, hypertension, and diabetes in Model 3. (Supplementary Tables 1–6 and Supplementary Figures 2–7).

### Subgroup analyses

**Fig. 2:**
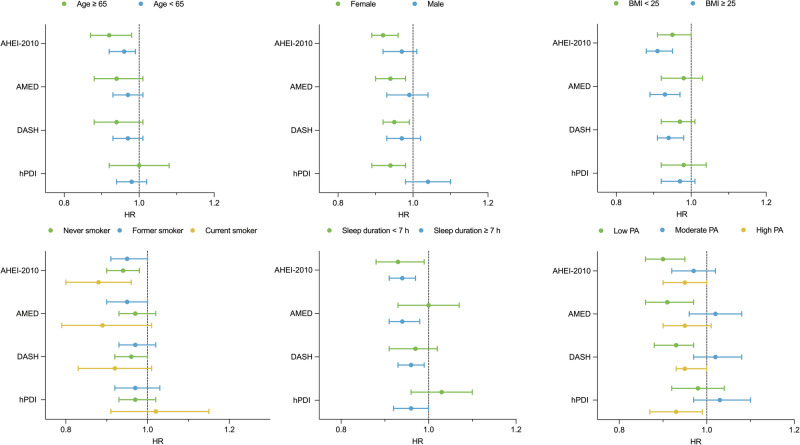
Associations between cumulative average dietary patterns (comparing the highest tertile with the lowest tertile) and major neuropsychiatric disorders in subgroups. Models were adjusted for age, sex, smoking status, alcohol consumption status, ethnicity, APOEε4, energy intake, education, sleep duration, physical activity, body mass index, and Townsend deprivation index. AMED Alternate Mediterranean Diet, AHEI-2010 Alternative Healthy Eating Index-2010, DASH Dietary Approaches to Stop Hypertension, hPDI healthful Plant-based Diet Index, HR hazard ratio.

The association between dietary patterns and major neuropsychiatric disorders persisted in subgroups defined by age, BMI, sex, smoking status, sleep duration and PA (Fig. 2). The inverse associations between the AHEI-2010, AMED, or DASH scores and dementia risk were strong in participants who were older, female, overweight or obese and low levels of PA. Although the hPDI scores were inversely associated with major neuropsychiatric disorders in women, no significant inverse association was observed in relation to age or body weight. Among current smokers, increased adherence to AHEI-2010 was associated with a reduced risk of developing neuropsychiatric disorders. In participants sleeping for >7 h, dietary patterns showed a strong inverse association with neuropsychiatric disorders. When stratified by PA, the inverse associations between healthy dietary patterns and major neuropsychiatric disorders were significant among participants with low PA, whereas no significant associations were observed in those with moderate PA. Overall, the protective associations of healthy dietary patterns with major neuropsychiatric disorders were generally consistent across subgroups, with stronger effects observed among older, female, overweight or obese participants, those with longer sleep duration and low levels of PA (Supplementary Table 7).

### Dietary patterns, inflammation, and metabolism

**Fig. 3:**
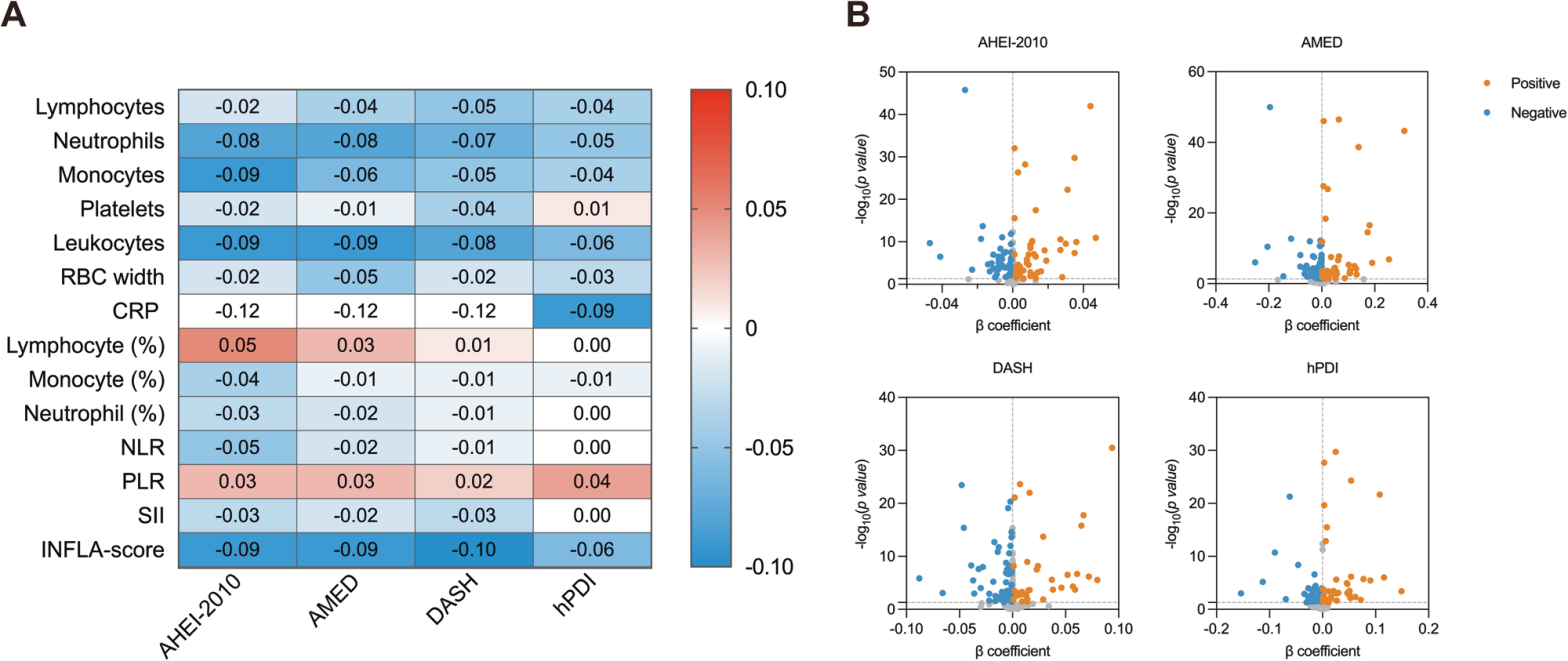
Associations between dietary patterns and circulating inflammatory or metabolic biomarkers. **A** Spearman correlation heatmap between four dietary pattern scores (AHEI-2010, AMED, DASH, and hPDI) and 14 inflammatory biomarkers, including absolute and percentage counts of immune cells, derived indices (NLR, PLR, SII), and the INFLA-score. Blue and red indicate negative and positive correlations, respectively. **B** Linear regression results show associations between dietary pattern scores and a comprehensive panel of metabolic biomarkers. Each dot represents a biomarker. Models were adjusted for age, sex, smoking status, alcohol consumption status, ethnicity, APOEε4, energy intake, education, sleep duration, physical activity, body mass index, and Townsend deprivation index. AMED Alternate Mediterranean Diet, AHEI-2010 Alternative Healthy Eating Index-2010, DASH Dietary Approaches to Stop Hypertension, hPDI healthful Plant-based Diet Index, RBC red blood cell, CRP C-reactive protein, NLR neutrophil-to-lymphocyte ratio, PLR platelet-to-lymphocyte ratio, SII systemic immune-inflammation index.

The correlations between dietary patterns and inflammation-related markers are shown in Fig. 3A. In this analysis, dietary patterns exhibited significant inverse associations with major proinflammatory biomarkers (β = -0.01 to -0.09) after adjusting for age, sex, ethnicity, BMI, Townsend deprivation index, PA, sleep, total energy intake, smoking status, alcohol consumption, education, and APOEε4 (Table 2). Several inflammation-related biomarkers showed inverse associations with all four dietary patterns (AHEI-2010, AMED, DASH, and hPDI). Neutrophils, leukocytes, C-reactive protein (CRP), and the INFLA-score exhibited the strongest inverse relationships with dietary patterns (β = -0.04 to -0.09). Additionally, monocytes were strongly associated with the AHEI-2010 and DASH scores, whereas red blood cell distribution width was strongly associated with the AMED and hPDI scores. Notably, platelets displayed a significant inverse association with the DASH scores (β = -0.04 to -0.08). After adjusting for the same covariates, most metabolic biomarkers were significantly associated with dietary patterns (Fig. 3B). The 10 metabolic biomarkers that were most strongly associated with dietary adherence were predominantly related to fatty acid metabolism (Supplementary Tables 8–11). Together, these findings indicate that healthy dietary patterns are associated with lower levels of systemic inflammation and favorable metabolic profiles.

**Table 2:** Associations between four healthy dietary patterns and inflammatory biomarkers

	AHEI-2010		AMED		DASH		hPDI	
Inflammation Indexes	β (95% CI)	*p*	β (95% CI)	*p*	β (95% CI)	*p*	β (95% CI)	*p*
Lymphocytes	-0.01 (-0.04, 0.01)	0.220	-0.02 (-0.05, 0.00)	0.046	-0.02 (-0.04, 0.00)	0.094	-0.03 (-0.06, 0.00)	0.024
Neutrophils	-0.07 (-0.09, -0.04)	<0.001	-0.06 (-0.08, -0.03)	<0.001	-0.05 (-0.08, -0.03)	<0.001	-0.05 (-0.07, -0.02)	0.001
Monocytes	-0.05 (-0.07, -0.02)	<0.001	-0.03 (-0.06, -0.01)	0.005	-0.04 (-0.07, -0.02)	<0.001	-0.03 (-0.05, 0.00)	0.049
Platelets	-0.02 (-0.05, 0.00)	0.050	-0.03 (-0.05, -0.01)	0.014	-0.04 (-0.06, -0.01)	0.003	0.00 (-0.02, 0.03)	0.731
Leukocytes	-0.07 (-0.09, -0.04)	<0.001	-0.06 (-0.08, -0.04)	<0.001	-0.06 (-0.08, -0.03)	<0.001	-0.06 (-0.08, -0.03)	<0.001
RBC width	-0.04 (-0.06, -0.02)	0.001	-0.06 (-0.08, -0.03)	<0.001	-0.03 (-0.06, -0.01)	0.007	-0.04 (-0.07, -0.02)	0.002
CRP	-0.06 (-0.08, -0.04)	<0.001	-0.06 (-0.08, -0.04)	<0.001	-0.06 (-0.08, -0.03)	<0.001	-0.04 (-0.07, -0.01)	0.003
Lymphocyte (%)	0.04 (0.02, 0.06)	0.001	0.02 (0.00, 0.05)	0.072	0.03 (0.00, 0.05)	0.020	0.01 (-0.02, 0.04)	0.452
Monocyte (%)	-0.01 (-0.03, 0.01)	0.456	0.00 (-0.02, 0.02)	0.977	-0.01 (-0.03, 0.01)	0.337	0.00 (-0.03, 0.03)	0.938
Neutrophil (%)	-0.03 (-0.05, -0.01)	0.014	-0.02 (-0.04, 0.01)	0.200	-0.02 (-0.04, 0.00)	0.087	0.00 (-0.03, 0.02)	0.827
NLR	-0.03 (-0.05, -0.01)	0.016	-0.01 (-0.04, 0.01)	0.302	-0.02 (-0.04, 0.01)	0.177	0.00 (-0.02, 0.03)	0.845
PLR	0.01 (-0.01, 0.03)	0.415	0.01 (-0.02, 0.03)	0.620	0.01 (-0.02, 0.03)	0.566	0.02 (-0.01, 0.05)	0.148
SII	-0.03 (-0.06, -0.01)	0.008	-0.02 (-0.05, 0.00)	0.091	-0.03 (-0.05, 0.00)	0.028	0.01 (-0.02, 0.04)	0.613
INFLA-score	-0.09 (-0.11, -0.06)	<0.001	-0.08 (-0.10, -0.06)	<0.001	-0.08 (-0.11, -0.06)	<0.001	-0.06 (-0.09, -0.04)	<0.001

### Mediation analyses of inflammation and metabolism

Given the observed link between dietary patterns and a low risk of neuropsychiatric disorders in the Cox analysis, we used structural equation models (SEMs) to explore the roles of inflammation and metabolism in this association. In the initial SEM models including multiple inflammatory markers, the overall model fit was suboptimal and the mediation effects of the INFLA-score and other markers were not statistically significant. In contrast, the pathways involving CRP were statistically significant. Therefore, we retained CRP as the representative inflammatory mediator to ensure model stability and interpretability (Supplementary Figures 8–10). In addition, we selected the ten metabolites most strongly associated with dietary patterns for inclusion in the SEM analysis. For AHEI-2010 and DASH patterns, the metabolites Triglycerides/Total Lipids in Medium very-low-density lipoprotein (VLDL) and Cholesterol/Total Lipids in Medium VLDL were both initially included but exhibited high collinearity. To ensure model stability, Triglycerides/Total Lipids in Medium VLDL was excluded from each, resulting in nine metabolites being retained in the final SEM analysis.

**Fig. 4:**
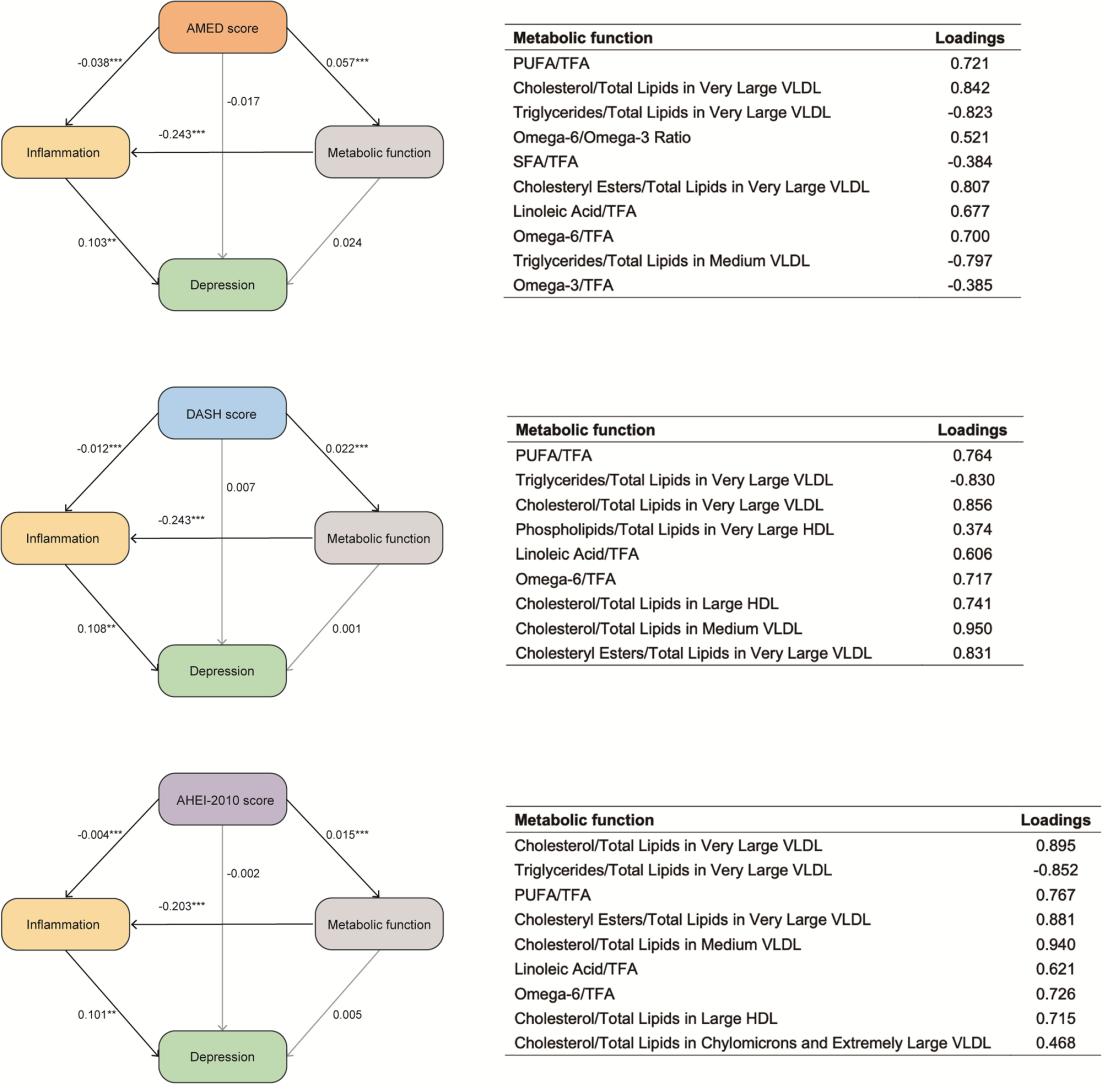
Structural equation models examining the associations of dietary pattern scores (Alternate Mediterranean Diet, Alternative Healthy Eating Index-2010, and Dietary Approaches to Stop Hypertension) with depression, mediated by inflammation and metabolic function. Standardized path coefficients are shown. AMED, DASH, and AHEI-2010 scores were significant predictors of inflammation (AMED: β = −0.038; DASH: β = −0.012; AHEI-2010: β = −0.004; all P < 0.001). Inflammation was a significant predictor of depression (AMED: β = 0.103, P = 0.004; DASH: β = 0.108, P = 0.002; AHEI-2010: β = 0.101, P = 0.001). Metabolic function was modeled as a latent variable using lipid-related metabolites and contributed to inflammatory pathways. All paths, except direct effects on depression, were statistically significant. No covariates were included in the models. *Two-sided unadjusted P < 0.05, **P < 0.01, ***P < 0.001. AMED Alternate Mediterranean Diet, AHEI-2010 Alternative Healthy Eating Index-2010, DASH Dietary Approaches to Stop Hypertension, VLDL very low-density lipoprotein, HDL high-density lipoprotein, TFA trans fatty acid, PUFA polyunsaturated fatty acid.

**Fig. 5:**
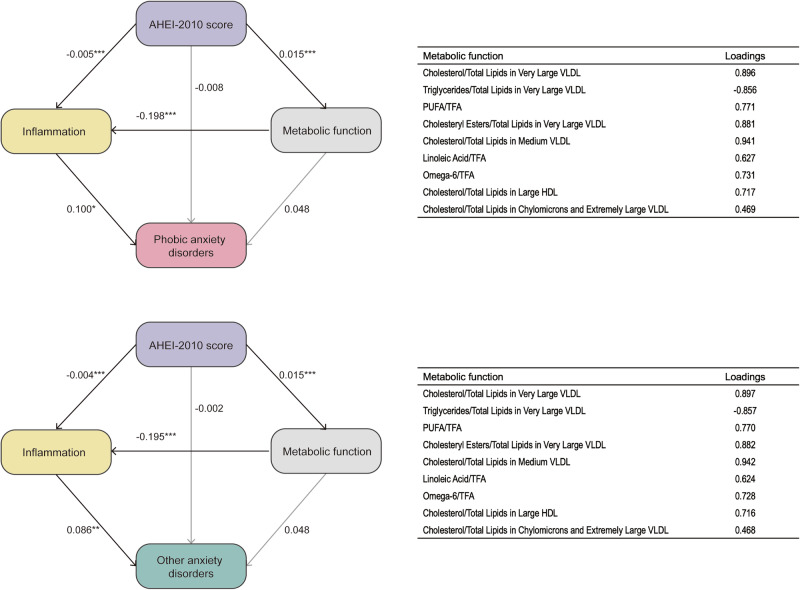
Structural equation models examining the associations between Alternative Healthy Eating Index-2010 dietary pattern and anxiety disorders, mediated by inflammation and metabolic function. Standardized path coefficients are shown. High AHEI-2010 scores were significantly associated with low levels of inflammation (β = −0.005 for phobic anxiety disorders; β = −0.004 for other anxiety disorders; both P < 0.001). Inflammation was positively associated with both types of anxiety (β = 0.100 and 0.086, respectively; both P < 0.05). Metabolic function was modeled as a latent variable composed of nine lipid-related biomarkers, contributing indirectly to the inflammation-anxiety pathway. No covariates were included in the models. *Two-sided unadjusted P < 0.05, **P < 0.01, ***P < 0.001. AHEI-2010 Alternative Healthy Eating Index-2010, VLDL very low-density lipoprotein, HDL high-density lipoprotein, TFA trans fatty acid, PUFA polyunsaturated fatty acid.

The results demonstrated a significant mediating effect of inflammation on the association between dietary patterns and depression. These results indicate that the AMED and DASH dietary patterns exert a direct anti-inflammatory effect, which subsequently contributes to the reduced risk of depression. In both models, high AMED, DASH, and AHEI-2010 scores were significantly correlated with low inflammation levels (AMED: β = -0.038, P < 0.001; DASH: β = -0.012, P < 0.001; AHEI-2010: β = -0.004, P < 0.001). Additionally, inflammation was a significant predictor of depression (AMED: β = 0.103, P = 0.004; DASH: β = 0.108, P = 0.002; AHEI: β = 0.101, P = 0.001). Furthermore, the results suggested that AMED, DASH, and AHEI-2010 dietary patterns regulate inflammation by influencing metabolism (AMED: β = -0.243, P < 0.001; DASH: β = -0.243, P < 0.001; AHEI-2010: β = -0.203, P < 0.001), thereby impacting the risk of depression. This highlights the potential role of diet-induced metabolic changes in modulating inflammation and mental health outcomes (Fig. 4). The AHEI-2010 dietary pattern was associated with a reduced risk of anxiety through the direct attenuation of inflammation (β = -0.005 and -0.004, P < 0.001) and indirect modulation of metabolic function to influence inflammatory responses (β = -0.198 and -0.195, P < 0.001). These findings suggest that inflammation and metabolically driven inflammatory processes may represent key pathways that link dietary patterns to the low risk of anxiety (Fig. 5).

Overall, dietary patterns may reduce the risk of neuropsychiatric disorders both by directly modulating inflammation and by regulating inflammation through metabolic pathways.

### Sensitivity analyses

In sensitivity analyses excluding individuals who developed the outcomes within the first 2 years of follow-up, the associations between diet quality scores and most mental health and dementia outcomes remained statistically significant. However, the associations between the DASH score and depression and between the AMED score and all-cause dementia were attenuated and no longer statistically significant (Supplementary Tables 12–17).

Furthermore, causal mediation analysis showed similar findings regarding the mediation effect (Supplementary Fig.11).

## Discussion

To date, most studies on the relationship between dietary patterns and neurodegenerative or psychiatric disorders have focused on a single disease outcome, with limited research integrating multiple outcomes under a unified biological mechanism. In this study, we comprehensively evaluated the association between several healthy dietary patterns and the risk of various neurological and psychiatric conditions, including all-cause dementia, AD, VaD, depression, and anxiety (Supplementary Table 18, Supplementary Fig.12). We observed that higher adherence to healthy dietary patterns was associated with significantly lower risk of neuropsychiatric disorders across multiple models, suggesting a protective effect of diet on brain health. Based on these findings, we explored the mediating roles of systemic inflammation and metabolic biomarkers. The results revealed significant associations between dietary patterns and inflammation levels and suggested that both inflammation and metabolic status partially mediate the relationship between diet and neuropsychiatric outcomes. Although the overall mediating effects were modest, these findings support a potential mechanistic model linking diet, inflammation and metabolism, and brain health.

In this study, adherence to the AHEI-2010 was significantly associated with a low risk of depression and anxiety, consistent with previous studies supporting its protective role in mental health^21,22^. The protective effect of AHEI-2010 appeared stronger for phobic anxiety disorders than for other anxiety disorders, which may be explained by its emphasis on antioxidant-rich foods that reduce oxidative stress and inflammation, potentially modulating amygdala hyperactivation, whereas prefrontal-limbic dysregulation underlying other anxiety disorders may be less responsive to dietary modulation^23,24^. AMED showed a significant inverse association with all-cause dementia; however, no statistically significant associations were found when examining AD and VaD separately, indicating partial inconsistency with previous findings^25,26^. These findings may reflect pathophysiological differences. AD is characterized by β-amyloid plaques and tau-related neurofibrillary tangles^27^, whereas VaD is mainly driven by hypertension- and atherosclerosis-related small vessel disease leading to chronic ischemia, blood-brain barrier dysfunction, and white matter disruption^28^. Although AMED emphasizes unsaturated fats (including omega-3 fatty acids) and polyphenols with anti-inflammatory and antioxidant properties, these nutrients may have limited impact on AD-specific pathology and are unlikely to sufficiently modify the vascular mechanisms underlying VaD. Additionally, increased adherence to AMED was associated with a low risk of depression^29^. Notably, this study provides novel evidence suggesting a potential inverse relationship between adherence to AMED and phobic anxiety. Although previous studies have primarily linked the DASH dietary pattern to reductions in depressive symptoms^30^, its broad role in mental health remains unclear. Our findings extend this evidence by demonstrating associations between adherence to DASH and a low risk of depression and anxiety. In contrast, no significant associations were observed between adherence to hPDI and mental health outcomes.

Notably, the AHEI-2010, DASH, and hPDI scores were not significantly associated with dementia risk in our analysis. Nevertheless, previous studies have shown that high adherence to AMED is associated with large hippocampal volume^31^ and that hPDI may be linked to reduced risks of dementia and depression^32^, although some evidence suggests that this protective effect may be limited to males or APOEε4 carriers. Regarding DASH, there is currently a lack of direct evidence supporting its association with reduced dementia risk. Combining the findings of the associations between the four dietary patterns and the risk of dementia, depression, and anxiety, our results support the potential protective effect of overall healthy dietary patterns on mental and psychological health. These dietary patterns share a core foundation of plant-based foods and whole grains, but they differ in specific components and targeted health outcomes. The AHEI-2010 emphasizes balancing various nutrients, with strict scoring for factors such as trans fat restriction, and is most strongly associated with promoting healthy aging as well as maintaining overall physical function and psychological well-being^33^. The AMED highlights the intake of fish and seafood, legumes, and nuts, while also emphasizing moderate alcohol consumption, which may exert beneficial effects on brain health by attenuating neuroinflammation, improving neurotransmitter metabolism, and promoting healthy brain development^34^. Despite its cardiovascular origins, the DASH diet conferred modest benefits against depressive symptoms, potentially via vascular-metabolic pathways^35^, which warrants further investigation in future studies. Collectively, these heterogeneous findings may be explained by the distinct dietary components emphasized in each pattern, differences in scoring algorithms, and the underlying pathophysiological mechanisms of disease subtypes. Additionally, the loss of significance in the associations between DASH and depression and between AMED and all-cause dementia in sensitivity analyses may partly reflect reverse causality. Moreover, the protective effects of dietary patterns may require longer follow-up to become evident, consistent with the cumulative nature of dietary influences on mental and cognitive health. In particular, the attenuation after excluding early cases suggests that baseline dietary characteristics may have been influenced by subclinical disease in individuals who developed outcomes within the first 2 years, and that the protective effects of DASH are therefore more relevant to primary prevention rather than secondary prevention contexts.

The AMED, AHEI-2010, DASH, and hPDI dietary patterns emphasize a high intake of plant-based foods, including vegetables, fruits, whole grains, legumes, seeds, and nuts. These foods are rich in anti-inflammatory and antioxidant compounds, such as flavonoids, polyphenols, and vitamins C and E. Long-term flavonoid intake has been associated with a low risk of AD and related dementia, primarily through improvements in memory, attention, and executive functioning^36^. Additionally, flavonoid consumption has been linked to reduced depressive symptoms and dementia risk, with the strongest protective effects observed among individuals with high genetic risk, hypertension, or preexisting depressive symptoms^37^. Seeds and nuts are rich in omega-3 fatty acids, especially alpha-linolenic acid, which has strong anti-inflammatory properties. These fatty acids may contribute to neuroprotection by suppressing proinflammatory cytokine production and modulating neuroinflammatory processes. Oxidative stress plays a pivotal role in the pathogenesis and mechanisms of dementia, which causes neuronal damage^38–40^. Given that oxidative stress is a key driver of inflammation and is strongly implicated in neuronal damage, brain aging, neurodegenerative diseases, and mood disorders, dietary antioxidants—such as vitamins C and E and polyphenols—may play a vital role in preserving cognitive function and preventing neurodegeneration^41^. Additionally, different dietary patterns have been shown to shape distinct gut microbiota compositions. Healthy dietary interventions, such as the Mediterranean diet, can enrich the gut microbiota and, through modulation of the microbiota–gut–brain axis, contribute to improvements in cognition and emotional regulation^42^.

However, other essential neuroprotective nutrients—such as vitamins B and D—are predominantly found in animal-sourced foods, including lean meat, eggs, dairy products, fish, and seafood. High intake of vitamin B has been inversely associated with stress-related symptoms and may protect against depression and anxiety^43^. Similarly, vitamin D, which is involved in cell growth, neuromuscular function, and immune regulation, has been associated with a low risk of major depressive disorders and anxiety^44,45^. Most clinical trials have suggested that vitamin D supplementation alleviates symptoms of depression and anxiety^46^. Moreover, minerals and trace elements are essential for the survival and growth of some gut bacteria, while deficiencies or toxicities promote the enrichment of pathogenic bacteria that trigger gut inflammation^42^.Therefore, the limited intake of animal-derived foods in plant-based diets, particularly strict hPDI, may contribute to the lack of significant associations with reduced risks of dementia, depression, and anxiety observed in this study. However, when analyzing the correlations of hPDI with inflammatory and metabolic markers, hPDI was inversely associated with most inflammatory indicators and showed significant correlations with the lipid composition of VLDL, but these potential benefits may have been offset. Additionally, dietary ω-3 PUFAs from fish consumption are associated with higher cognitive function and exert protective effects against depression^47,48^. These findings highlight the potential limitations of strict plant-based dietary patterns for providing sufficient neuroprotective nutrients. However, vegetable oils, which were not available in the UKB database, may have anti-inflammatory properties, and this requires further investigation.

Moreover, several studies have shown that the consumption of red and processed meat is associated with altered serum lipid levels, such as triglycerides, and positively correlated with higher serum CRP levels^49–51^. Furthermore, ultra-processed foods are recognized as triggers of low-grade systemic inflammatory and oxidative changes^52,53^. Chronic low-grade inflammation is a well-established contributor to the development of cardiovascular diseases, diabetes, neurodegenerative disorders, and mood-related conditions^54–56^. Consequently, excessive and long-term consumption of processed meat may increase the risk of these health outcomes. Similarly, frequent consumption of sweets, including cakes, candies, and sugar-sweetened beverages, can result in rapid spikes in blood glucose levels, increased insulin secretion, and fat accumulation, which may trigger oxidative stress and systemic inflammation^57^. This may partially explain their detrimental associations with cognitive and mental health outcomes.

These mechanistic insights are largely consistent with the associations observed between healthy dietary pattern adherence and a reduced risk of neurocognitive and mood disorders. In this study, which was grounded in a unified biological framework, we systematically examined the interrelationships among diet, inflammation, and common neuropsychiatric disorders. Our findings support the hypothesis that dietary patterns influence systemic inflammation primarily by modulating metabolic function. Several healthy dietary patterns were significantly associated with low levels of multiple circulating inflammatory markers, indicating their potential anti-inflammatory effects. In the mediation analysis, we initially incorporated multiple inflammatory indicators, including CRP, INFLA-score, neutrophil-to-lymphocyte (NLR) and platelet-to-lymphocyte (PLR), but the overall model fit was poor, and only CRP exhibited a consistent and statistically significant mediating effect. This may reflect the sensitivity of CRP in detecting chronic systemic inflammation, which has been implicated in triggering or exacerbating neuroinflammatory processes. The observed inverse association between dietary patterns and CRP levels may be partly explained by the high intake of plant-based foods, such as vegetables and fruits, which are rich in anti-inflammatory and antioxidant compounds.

Additionally, we observed that long-term adherence to healthy dietary patterns may influence neuropsychiatric outcomes by improving metabolic function. Previous prospective studies have consistently identified metabolic dysregulation, such as impaired glucose metabolism and dyslipidemia, as the major risk factor for dementia, depression, and anxiety^58–60^. Our results suggest that diet-induced metabolic changes may contribute to disease prevention. Notably, we found a strong correlation between metabolic and inflammatory profiles, indicating that diet-driven alterations in small-molecule metabolites may influence inflammatory signaling pathways, thereby exerting either proinflammatory or anti-inflammatory effects and ultimately affecting the risk of neuropsychiatric disorders. In our analysis, several metabolites and pathways appeared to play a central role in this process, including the composition of lipids in very large VLDL particles — specifically, the ratios of cholesterol and cholesteryl esters to total lipids and triglycerides to total lipids — and the overall proportion of polyunsaturated fatty acids (PUFAs). The lipid composition of very large VLDL particles has been linked to insulin resistance, type 2 diabetes, and coronary artery calcification, all of which are known to contribute to the development of cognitive impairment and mental disorders^61^. Among the PUFAs, omega 3 polyunsaturated fatty acids (n-3 PUFAs) obtained from the diet are essential. They exert anti-inflammatory effects and modulate lipid metabolism by inhibiting inflammatory signaling pathways, such as nuclear factor-κB (NF-κB), down-regulating fatty acid synthesis via sterol regulatory element binding protein-1c (SREBP-1c), and up-regulating fatty acid oxidation through peroxisome proliferator-activated receptor α (PPARα). In addition, the incorporation of n-3 PUFAs into cell membrane phospholipids enhances membrane fluidity, which can lead to modifications in the way transmembrane proteins, such as receptors, interact with their ligands (Supplementary Tables 19–21, Supplementary Figure 13)^62,63^.

The biological pathways explored in this study provide additional evidence of the protective role of diet in common age-related neuropsychiatric conditions. Our findings deepen our current understanding of the diet-metabolism-inflammation-disease axis and offer a theoretical foundation for preventive strategies based on nutritional interventions. Given that dietary patterns are modifiable and sustainable lifestyle factors, beneficial patterns (such as AMED, AHEI-2010, and DASH) may inform personalized dietary recommendations, the development of public health nutrition guidelines, and multidisciplinary intervention strategies targeting inflammation and metabolic regulation. These strategies include population-level dietary guidelines, community-based education programs, and personalized nutrition approaches that consider genetic and lifestyle risk factors. Moreover, at the clinical level, integrating dietary counseling into routine care may further facilitate early prevention among high-risk individuals. Furthermore, this study highlights the importance of specific neuroprotective nutrients in maintaining cognitive and mental health and offers potential directions for early screening and supplementation strategies in older populations.

This study has some limitations. First, dietary patterns are not static and may change over time owing to factors such as demographic location, environmental influences, lifestyle modifications, or major health events. Meanwhile, although the cultural adaptability of the AMED score has been internationally recognized, the inclusion of alcohol consumption may not be appropriate in non-Mediterranean regions, highlighting the need for future studies to explore localized dietary scoring systems that better reflect cultural dietary practices. Second, both diet-induced metabolic alterations and chronic inflammation are cumulative processes that typically evolve over long periods. Therefore, long-term follow-up may be needed to fully capture their long-term effects. Additionally, dietary pattern scores were derived using tertile-based categorization. While this approach facilitates group comparisons, it may not fully reflect actual dietary intake levels, potentially underestimating inter-individual variability and limiting the assessment of potential dose-response relationships. Although our study included a broad range of metabolomic and inflammatory biomarkers across multiple pathways, these markers may not capture the complexity of the body’s metabolism-inflammatory network. Finally, as the study population primarily consisted of middle-aged and older adults, the generalizability of the findings to younger or more diverse populations warrants further investigation.

In conclusion, adherence to healthy dietary patterns—particularly AMED and AHEI-2010—was associated with lower risks of dementia, depression, and anxiety. The associations between dietary patterns and neuropsychiatric outcomes are likely mediated through inflammatory and metabolic pathways, highlighting the importance of precision-targeted dietary recommendations that modulate the diet-immune-brain axis. Our findings suggest that adherence to healthy dietary patterns could represent a promising public health strategy for the prevention of neuropsychiatric disorders. However, further research, including interventional studies, is needed to confirm causal relationships and clarify the underlying mechanisms.

## Methods

### Study population

The UKB is a prospective cohort study that enrolled over 500,000 participants aged 37–73 years from 22 assessment centers between 2006 and 2010 from England, Scotland, and Wales^64^. The UKB study was approved by the National Information Governance Board for Health and Social Care and the National Health Service North West Centre for Research Ethics Committee (ref. ^21^/NW/0157). All the participants provided their informed consent using an electronic signature system. In the UKB study, five dietary assessments were performed, including a baseline interview from 2006 to 2010 and four online 24 h diet recall questionnaires administered between February and April, June and August, and October and December 2011 as well as between April and June 2012. A total of 210,882 participants completed at least one online 24-h diet recall questionnaire.

### Dietary assessment

Diet was obtained using an online 24 h food intake assessment system^65^. The questionnaire first assessed whether the respondent had consumed a specific food category in the past 24 h. If the answer was yes, the intake of specific foods and their portion sizes was inquired. In this study, we set the most recent assessment date as the baseline and used the average intake derived from multiple completed dietary questionnaires as the baseline dietary measure.

### Alternate healthy eating index-2010

The AHEI-2010 evaluates diet quality based on adherence to U.S. federal dietary guidance aimed at reducing the risk of chronic diseases^66^. The index includes 11 dietary components, each scored from 0 to 10, resulting in a total score ranging from 0 to 110. Specific components, including fish (representing long-chain omega-3 fatty acids) and salt added to food (representing sodium), are detailed in the Supplementary Table 22.

### Alternate Mediterranean diet

The AMED is an adaptation of the traditional Mediterranean diet for non-Mediterranean diet countries. AMED scores were calculated based on the nine food groups. Participants received a score of 1 for intakes above the sex-specific median in the following categories: whole grains, vegetables (excluding potatoes), fruits, nuts and seeds, legumes, fish, and the ratio of monounsaturated to saturated fatty acids. All other intake levels were assigned a score of 0. For red and processed meat, those with an intake below the median received a score of 1. Alcohol consumption within the range of 5–15 g/day was assigned a score of 1. The total AMED score ranged from 0 to 9, with higher scores indicating a healthier diet^67^ (Supplementary Table 23).

### Dietary Approaches to Stop Hypertension

The DASH is a well-established dietary strategy for controlling blood pressure and reducing cardiovascular risk. It specifies high consumption of plant-based foods and limited intake of saturated fatty acids, total fat, cholesterol, and sodium. Each food group was categorized into quintiles according to sex. Scores were assigned in a positive direction for beneficial food groups (fruits, vegetables, nuts and legumes, whole grains, and low-fat dairy), with increasing quintiles receiving scores ranging from 1 to 5. Conversely, scores were assigned in the reverse direction for harmful food groups (sodium, red and processed meat, and sugar-sweetened beverages), with increasing quintiles receiving scores ranging from 5 to 1^68^ (Supplementary Table 24).

### Healthful plant-based diet index

The hPDI is designed to assess adherence to a plant-based diet, which involves minimizing or eliminating the intake of eggs, dairy, fish, and meat to reduce the risk of major chronic diseases^13^. Scores of 1–5 were assigned to quintiles of 17 food groups using inverse scoring by category: plant-based foods (whole grains, vegetables, fruits, nuts, legumes, tea/coffee) were awarded 5 points for the highest quintile and 1 for the lowest; conversely, animal-based foods (animal fats, dairy, eggs, fish/seafood, meat, other animal foods) were assigned 1 point at the highest quintile and 5 at the lowest; while other foods (refined grains, potatoes, sugar-sweetened beverages, fruit juice, sweets/desserts) received 1 point exclusively to the highest quintile, yielding a total score range of 17–85 points. We did not include vegetable oils in this study because they were not available in the UKB database^69^ (Supplementary Table 25).

### Covariate assessment

We considered sex, age, ethnicity, smoking status, alcohol frequency, qualification (education), physical activity (PA), body mass index (BMI), sleep duration, APOEε4 genotype, and Townsend deprivation index as covariates. The Townsend deprivation index was used to categorize socioeconomic status based on national census data for each postal code area. In addition, we included the average daily total energy intake, history of cardiovascular and cerebrovascular disease, hypertension, and diabetes in the models.

### Outcome definition

The outcomes were major neuropsychiatric diseases, including all-cause dementia, depression, phobic anxiety disorders, and other anxiety disorders. The secondary outcomes for all-cause dementia were AD and VaD. These diseases were assessed using self-reported medical conditions, primary care, hospital data, and death registers^70^. Outcome events were defined according to the International Classification of Diseases, Tenth Revision and are listed in Supplementary Table 26. Participant follow-up extended from baseline assessment completion until the earliest occurrence of: study outcome, death, or study end date (December 31, 2022).

### Blood inflammatory markers and metabolomics

At baseline (2006–2010), blood biomarker data were collected, and high-throughput nuclear magnetic resonance-based metabolomic analyses of a subset of blood samples were performed. The subsequent round of sample collection was conducted between 2012 and 2013. Our study included data from the 2012–2013 sample collection period. Blood inflammatory markers included lymphocytes, neutrophils, monocytes, platelet counts, leukocytes, red blood cell distribution width (RBC width), CRP, lymphocyte percentage, monocyte percentage, and neutrophil percentage. We additionally computed three hematologic ratios and a composite score as inflammatory markers: NLR, PLR, systemic immune-inflammation index (SII), and INFLA-score. The INFLA-score, a useful tool for assessing low-grade inflammation, was computed based on four components (CRP level, leukocyte count, platelet count, and NLR)^71^. The INFLA-score ranged from −16 to +16^72^. We quantified 251 metabolites across key metabolic pathways: 14 lipoprotein subclasses, fatty acids (including compositional profiles), and diverse low-molecular-weight species.

### Statistical analysis

We used tertiles of dietary scores to represent baseline characteristics. Continuous variables are presented as mean ± standard deviation, whereas categorical variables are expressed as frequency (percentage).

Cox proportional hazards regression models were used to examine the association between the four dietary patterns and several neuropsychiatric diseases, with adjustment for potential confounding variables. We calculated the hazard ratios (HRs) and the corresponding 95% confidence intervals (CIs) for this analysis. Model 1 was adjusted for age, sex, and total energy intake. Model 2 was adjusted for age, sex, smoking status, alcohol consumption, ethnicity, total energy intake, education, sleep duration, PA, BMI, APOEε4 genotype, and Townsend deprivation index. Model 3 was further adjusted for baseline medical history of diabetes and cardiovascular diseases. We analyzed dietary scores as both categorical (tertiles) and continuous (each quintile increment) variables. Furthermore, all analyses were stratified according to age, sex, BMI, smoking status, sleep duration and PA.

Subsequently, multiple linear regression models were used to assess the association of the four dietary scores with each inflammatory marker and metabolite. SEMs were used to examine the mediating roles of inflammation and metabolic function in the relationship between dietary patterns and neuropsychiatric diseases (implemented in the R package lavaan). The latent variable representing metabolic function was calculated using the first 10 significant markers that correlated with dietary patterns. Before analysis, inflammatory and metabolic biomarkers were standardized.

Finally, a sensitivity analysis was performed. We examined the association between dietary pattern scores and the risk of individual diseases by excluding individuals who developed the corresponding disease during the first 2 years of follow-up. We further utilized another R package (mediation) to perform a causal mediation analysis to examine whether inflammation mediated the association between dietary patterns and these outcomes.

Statistical analyses were performed using the R software (version 4.4.1). We report unadjusted P*-*values based on two-sided statistical tests, and P < 0.05 was considered statistically significant. All hazard ratios are expressed with their corresponding 95% confidence intervals in the format HR (95% CI).

## Supplementary information

Supplementary data

## Data Availability

For the data of UK Biobank, please visit https://www.ukbiobank.ac.uk/.

## Code availability

No custom code or mathematical algorithm was developed for this study.
