# Supplementary material for: Healthy dietary patterns for prevention of neuropsychiatric disorders: role of inflammatory and metabolic mechanisms
Source: NPJ Sci Food. 2025 Dec 9;9:264. doi: 10.1038/s41538-025-00623-4 (PMC12689847; doi:10.1038/s41538-025-00623-4)
Supplement: Supplementary file 1 — Supplementary data [file 41538_2025_623_MOESM1_ESM.pdf]

Healthy dietary patterns for prevention of neuropsychiatric disorders: Role of inflammatory and metabolic mechanisms_ Supplementary materials

Table of Contents

1. Participant Selection Flowchart---3

2. Cox Regression Results--4

3. Hazard ratios by diet quality scores---10

4. Stratified analyses of dietary patterns and outcomes--------------------------16

5. Dietary Patterns and Metabolic Biomarkers------------------------------------17

6. Results and Goodness-of-fit indices of SEM-----------------------------------19

7. Results of Sensitivity Analyses--22

8. Results of Mediation Analyses--28

9. Components and Scoring Criteria of Dietary Patterns------------------------29

10. Conceptual framework of the study--30

11. Interaction effects between dietary pattern and metabolic markers----------31

12. Dietary patterns and metabolic mediating pathway---------------------------34

13. Dietary Patterns Scoring in UK Biobank--------------------------------------35

14. ICD-10 Codes for Major Neuropsychiatric Disorders------------------------39

- **Supplementary Figure 1.** Flowchart of selection of this study participants included in analysis.

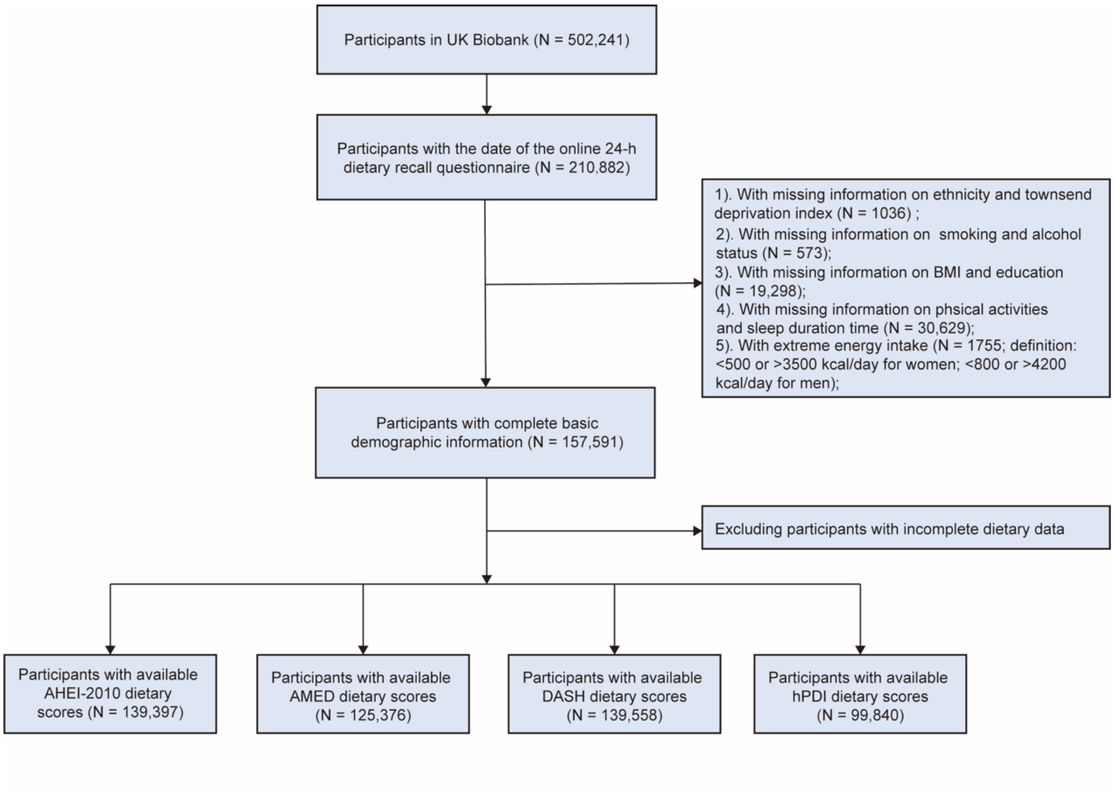

Abbreviations: AHEI, Alternative Healthy Eating Index; AMED, alternate Mediterranean diet; DASH, Dietary Approaches to Stop Hypertension; hPDI, Healthful plant-based diet index; BMI, body mass index

- **Supplementary Table 1.** Hazard ratios (95% confidence interval) of all-cause dementia according to tertiles or 20 percentile increase in adherence to diet quality scores in the UK Biobank cohort

Dietary pattern quality	Tertile1	Tertile2	Tertile3	Per 20-percentiles	p-trend
AMED, range	0, 3	4, 5	6, 9		
n/N	539/55015	451/48228	215/22053	1205/125296	
Model1	Ref.	0.866(0.764, 0.982)	0.851(0.725, 0.998)	0.967(0.929, 1.008)	0.020
Model2	Ref.	0.877(0.773, 0.995)	0.862(0.733, 1.014)	0.972(0.933, 1.013)	0.035
Model3	Ref.	0.878(0.774, 0.996)	0.857 (0.729, 1.008)	0.972(0.932, 1.013)	0.030
AHEI, range	8.843, 43.623	43.623, 54.333	54.334, 97.500		
n/N	435/46439	455/46447	452/46429	1342/139315	
Model1	Ref.	0.908(0.795, 1.036)	0.850(0.744, 0.972)	0.952(0.916, 0.989)	0.018
Model2	Ref.	0.930(0.814, 1.062)	0.883(0.770, 1.011)	0.963(0.926, 1.001)	0.073
Model3	Ref.	0.934(0.817, 1.066)	0.884(0.771, 1.013)	0.964(0.927, 1.002)	0.077
DASH, range	8, 22	23, 26	27, 40		
n/N	439/52474	432/43696	471/43306	1342/139476	
Model1	Ref.	0.916(0.802, 1.046)	0.884(0.776, 1.008)	0.977(0.940, 1.015)	0.068
Model2	Ref.	0.927(0.811, 1.060)	0.898(0.786, 1.026)	0.985(0.947, 1.024)	0.117
Model3	Ref.	0.930(0.813, 1.063)	0.897(0.785, 1.025)	0.985(0.947, 1.024)	0.112
hPDI, range	42, 57	58, 61	62, 78		
n/N	310/35665	393/37865	283/26249	986/ 99779	
Model1	Ref.	1.004(0.863, 1.167)	0.958(0.811, 1.131)	0.979(0.935,1.024)	0.615
Model2	Ref.	1.014(0.871, 1.179)	0.962(0.813, 1.137)	0.980(0.936, 1.026)	0.651
Model3	Ref.	1.005(0.863, 1.169)	0.963(0.815, 1.138)	0.979(0.935, 1.025)	0.663

- **Supplementary Table 2.** Hazard ratios (95% confidence interval) of Alzheimer's disease according to tertiles or 20 percentile increase in adherence to diet quality scores in the UK Biobank cohort

Dietary pattern quality	Tertile1	Tertile2	Tertile3	Per 20-percentiles	p-trend
AMED, range	0, 3	4, 5	6, 9		
n/N	218/55043	200/48263	109/22065	527/125371	
Model1	Ref.	0.944(0.779, 1.145)	1.051(0.833, 1.326)	1.015(0.953, 1.080)	0.821
Model2	Ref.	0.939(0.774, 1.140)	1.020(0.806, 1.291)	1.008(0.946, 1.073)	0.997
Model3	Ref.	0.940(0.774, 1.141)	1.016(0.803, 1.286)	1.007(0.945, 1.073)	0.975
AHEI, range	8.843, 43.623	43.623, 54.333	54.334, 97.500		
n/N	169/46452	204/46457	209/46445	582/139354	
Model1	Ref.	1.022(0.833, 1.254)	0.963(0.784, 1.184)	0.992(0.936, 1.051)	0.698
Model2	Ref.	1.017(0.827, 1.249)	0.947(0.768, 1.167)	0.985(0.928, 1.045)	0.583
Model3	Ref.	1.019(0.830, 1.252)	0.946(0.767, 1.167)	0.985(0.929, 1.045)	0.578
DASH, range	8, 22	23, 26	27, 40		
n/N	167/52487	177/43706	238/43322	582/139515	
Model1	Ref.	0.978(0.792, 1.209)	1.163(0.953, 1.419)	1.055(0.994, 1.119)	0.113
Model2	Ref.	0.953(0.770, 1.179)	1.116(0.912, 1.366)	1.044(0.983, 1.109)	0.242
Model3	Ref.	0.955(0.772, 1.182)	1.115(0.911,1.364)	1.044(0.983, 1.109)	0.247
hPDI, range	42, 57	58, 61	62, 78		
n/N	127/35534	162/37837	136/26440	425/99811	
Model1	Ref.	1.001(0.791, 1.267)	1.096(0.854, 1.407)	0.997(0.930, 1.069)	0.466
Model2	Ref.	0.998(0.788, 1.264)	1.074(0.835, 1.381)	0.990(0.923, 1.063)	0.575
Model3	Ref.	1.001(0.790, 1.268)	1.072(0.833, 1.379)	0.990(0.922, 1.062)	0.583

- **Supplementary Table 3.** Hazard ratios (95% confidence interval) of Vascular dementia according to tertiles or 20 percentile increase in adherence to diet quality scores in the UK Biobank cohort

Dietary pattern quality	Tertile1	Tertile2	Tertile3	Per 20-percentiles	p-trend
AMED, range	0, 3	4, 5	6, 9		
n/N	93/55030	73/48244	44/22063	210/125336	
Model1	Ref.	0.810(0.596, 1.102)	1.010(0.703, 1.451)	0.991(0.898, 1.093)	0.770
Model2	Ref.	0.860(0.631, 1.171)	1.135(0.786, 1.639)	1.024(0.926, 1.131)	0.744
Model3	Ref.	0.863(0.633, 1.177)	1.123(0.778, 1.623)	1.023(0.926, 1.131)	0.771
AHEI, range	8.843, 43.623	43.623, 54.333	54.334, 97.500		
n/N	80/46451	72/46458	85/46448	237/139357	
Model1	Ref.	0.787(0.571, 1.083)	0.895(0.657, 1.221)	0.986(0.900, 1.080)	0.512
Model2	Ref.	0.835(0.605, 1.151)	0.992(0.724, 1.360)	1.019(0.929, 1.118)	0.978
Model3	Ref.	0.843(0.612, 1.163)	0.997(0.727, 1.367)	1.022(0.931, 1.121)	0.997
DASH, range	8, 22	23, 26	27, 40		
n/N	64/52486	84/43707	89/43325	237/139518	
Model1	Ref.	1.191(0.860, 1.650)	1.102(0.798, 1.522)	1.042(0.949, 1.144)	0.605
Model2	Ref.	1.272(0.916, 1.766)	1.215(0.875, 1.687)	1.079(0.981, 1.186)	0.274
Model3	Ref.	1.277(0.919, 1.773)	1.212(0.873, 1.683)	1.079(0.982, 1.186)	0.283
hPDI, range	42, 57	58, 61	62, 78		
n/N	50/35533	71/37838	50/26442	171/99813	
Model1	Ref.	1.156(0.801, 1.669)	1.105(0.738, 1.656)	1.035(0.926, 1.157)	0.625
Model2	Ref.	1.202(0.832, 1.738)	1.148(0.764, 1.727)	1.048(0.937, 1.172)	0.503
Model3	Ref.	1.221(0.845, 1.765)	1.150(0.765, 1.729)	1.047(0.937, 1.171)	0.498

- **Supplementary Table 4.** Hazard ratios (95% confidence interval) of depression according to tertiles or 20 percentile increase in adherence to diet quality scores in the UK Biobank cohort

Dietary pattern quality	Tertile1	Tertile2	Tertile3	Per 20-percentiles	p-trend
AMED, range	0, 3	4, 5	6, 9		
n/N	1616/50519	1271/44581	514/20477	3401/115577	
Model1	Ref.	0.884(0.821, 0.952)	0.764(0.691, 0.844)	0.922(0.900, 0.944)	<0.001
Model2	Ref.	0.944(0.876, 1.017)	0.867(0.783, 0.960)	0.955(0.933, 0.979)	0.005
Model3	Ref.	0.945(0.877, 1.018)	0.866(0.782, 0.958)	0.955(0.932, 0.979)	0.005
AHEI, range	8.843, 43.623	43.623, 54.333	54.334, 97.500		
n/N	1414/42566	1251/42829	1172/43096	3837/128490	
Model1	Ref.	0.847(0.784, 0.914)	0.755(0.698, 0.818)	0.910(0.889, 0.931)	<0.001
Model2	Ref.	0.924(0.856, 0.999)	0.874(0.806, 0.948)	0.952(0.931, 0.975)	0.001
Model3	Ref.	0.927(0.858, 1.001)	0.879(0.810, 0.953)	0.954(0.932, 0.976)	0.001
DASH, range	8, 22	23, 26	27, 40		
n/N	1625/48065	1104/40398	1113/40178	3842/128641	
Model1	Ref.	0.807(0.748, 0.872)	0.817(0.756, 0.883)	0.933(0.912, 0.954)	<0.001
Model2	Ref.	0.877(0.812, 0.948)	0.928(0.857, 1.004)	0.973(0.951, 0.996)	0.037
Model3	Ref.	0.880(0.815, 0.951)	0.933(0.862, 1.009)	0.975(0.953, 0.997)	0.051
hPDI, range	42, 57	58, 61	62, 78		
n/N	1031/32586	979/34954	737/24512	2747/92052	
Model1	Ref.	0.881(0.806, 0.963)	0.930(0.844, 1.026)	0.970(0.944, 0.996)	0.104
Model2	Ref.	0.922(0.843, 1.008)	1.001(0.907, 1.105)	0.992(0.966, 1.020)	0.877
Model3	Ref.	0.920(0.841, 1.006)	0.997(0.903, 1.101)	0.991(0.965, 1.019)	0.816

- **Supplementary Table 5.** Hazard ratios (95% confidence interval) of phobic anxiety disorders according to tertiles or 20 percentile increase in adherence to diet quality scores in the UK Biobank cohort

Dietary pattern quality	Tertile1	Tertile2	Tertile3	Per 20-percentiles	p-trend
AMED, range	0, 3	4, 5	6, 9		
n/N	244/54865	187/48100	76/21973	507/124938	
Model1	Ref.	0.824(0.681, 0.998)	0.688(0.530, 0.893)	0.895(0.841, 0.952)	0.003
Model2	Ref.	0.868(0.716, 1.052)	0.759(0.583, 0.988)	0.920(0.864, 0.979)	0.028
Model3	Ref.	0.869(0.717, 1.054)	0.757(0.581, 0.985)	0.870(0.768, 0.985)	0.027
AHEI, range	8.843, 43.623	43.623, 54.333	54.334, 97.500		
n/N	204/46312	189/46322	180/46277	573/138911	
Model1	Ref.	0.812(0.665, 0.991)	0.694(0.565, 0.851)	0.902(0.851, 0.956)	<0.001
Model2	Ref.	0.870(0.711, 1.062)	0.780(0.633, 0.961)	0.936(0.882, 0.994)	0.020
Model3	Ref.	0.872(0.714, 1.066)	0.784(0.636, 0.966)	0.937(0.883, 0.995)	0.022
DASH, range	8, 22	23, 26	27, 40		
n/N	238/52325	167/43579	169/43168	574/139072	
Model1	Ref.	0.770(0.639, 0.952)	0.762(0.624, 0.931)	0.909 (0.858, 0.963)	0.006
Model2	Ref.	0.833(0.681, 1.017)	0.844(0.689, 1.034)	0.940(0.887, 0.997)	0.088
Model3	Ref.	0.836(0.684, 1.021)	0.848(0.692, 1.039)	0.942(0.888, 0.999)	0.096
hPDI, range	42, 57	58, 61	62, 78		
n/N	148/35410	143/37711	97/26364	388/99485	
Model1	Ref.	0.825(0.653, 1.042)	0.736(0.565, 0.958)	0.919(0.855, 0.988)	0.021
Model2	Ref.	0.861(0.681, 1.088)	0.786(0.602, 1.027)	0.939(0.873, 1.010)	0.072
Model3	Ref.	0.861(0.681, 1.089)	0.785(0.601, 1.025)	0.939(0.873, 1.009)	0.070

- **Supplementary Table 6.** Hazard ratios (95% confidence interval) of other anxiety disorders according to tertiles or 20 percentile increase in adherence to diet quality scores in the UK Biobank cohort

Dietary pattern quality	Tertile1	Tertile2	Tertile3	Per 20-percentiles	p-trend
AMED, range	0, 3	4, 5	6, 9		
n/N	1910/52985	1483/46539	731/21246	4124/120770	
Model1	Ref.	0.867(0.810, 0.929)	0.911(0.836, 0.993)	0.952(0.932, 0.973)	0.003
Model2	Ref.	0.909(0.849, 0.974)	0.988(0.906, 1.079)	0.975(0.954, 0.997)	0.291
Model3	Ref.	0.910(0.850, 0.975)	0.988(0.905, 1.078)	0.975(0.954, 0.997)	0.291
AHEI, range	8.843, 43.623	43.623, 54.333	54.334, 97.500		
n/N	1658/44689	1491/44855	1494/44740	4643/134284	
Model1	Ref.	0.847(0.789, 0.909)	0.802(0.747, 0.862)	0.931(0.912, 0.950)	<0.001
Model2	Ref.	0.902(0.840, 0.968)	0.887(0.824, 0.954)	0.961(0.941, 0.981)	0.001
Model3	Ref.	0.904(0.842, 0.971)	0.890(0.828, 0.958)	0.962(0.942, 0.982)	0.002
DASH, range	8, 22	23, 26	27, 40		
n/N	1888/50532	1371/42193	1387/41713	4646/134438	
Model1	Ref.	0.862(0.804, 0.925)	0.875(0.816, 0.939)	0.957(0.938, 0.976)	<0.001
Model2	Ref.	0.909(0.847, 0.975)	0.945(0.879, 1.015)	0.982(0.962, 1.003)	0.089
Model3	Ref.	0.911(0.849, 0.978)	0.948(0.883, 1.019)	0.983(0.963, 1.004)	0.111
hPDI, range	42, 57	58, 61	62, 78		
n/N	1264/34221	1205/36456	861/25496	3330/96173	
Model1	Ref.	0.888(0.819, 0.962)	0.884(0.808, 0.967)	0.966(0.942, 0.990)	0.005
Model2	Ref.	0.914(0.844, 0.991)	0.928(0.848, 1.016)	0.981(0.957, 1.005)	0.079
Model3	Ref.	0.914(0.843, 0.990)	0.926(0.845, 1.013)	0.959(0.916, 1.003)	0.070

- **Supplementary Figure 2.** Hazard ratios (95% confidence interval) of all-cause dementia according to diet quality scores in the UK Biobank cohort

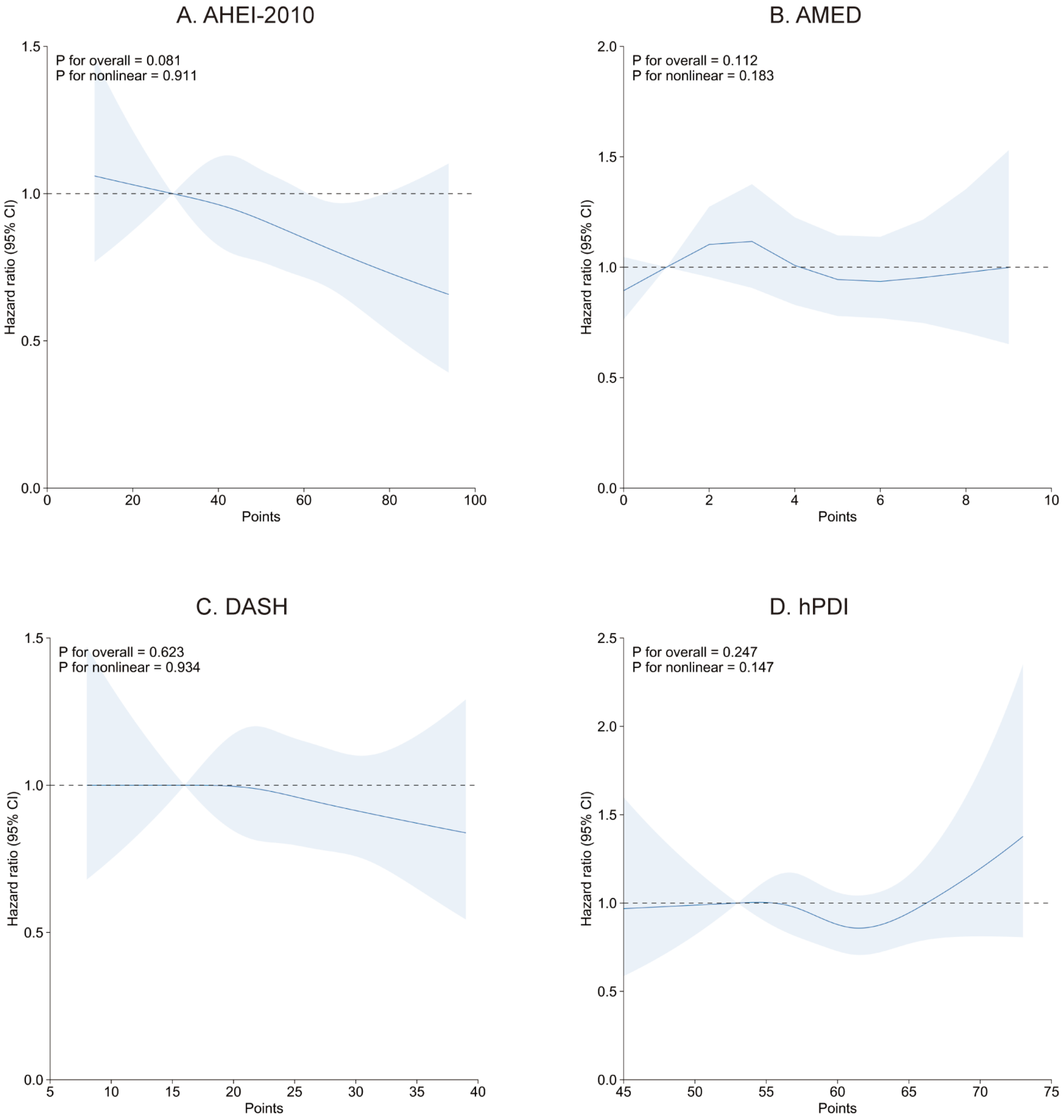

- **Supplementary Figure 3.** Hazard ratios (95% confidence interval) of Alzheimer's disease according to diet quality scores in the UK Biobank cohort

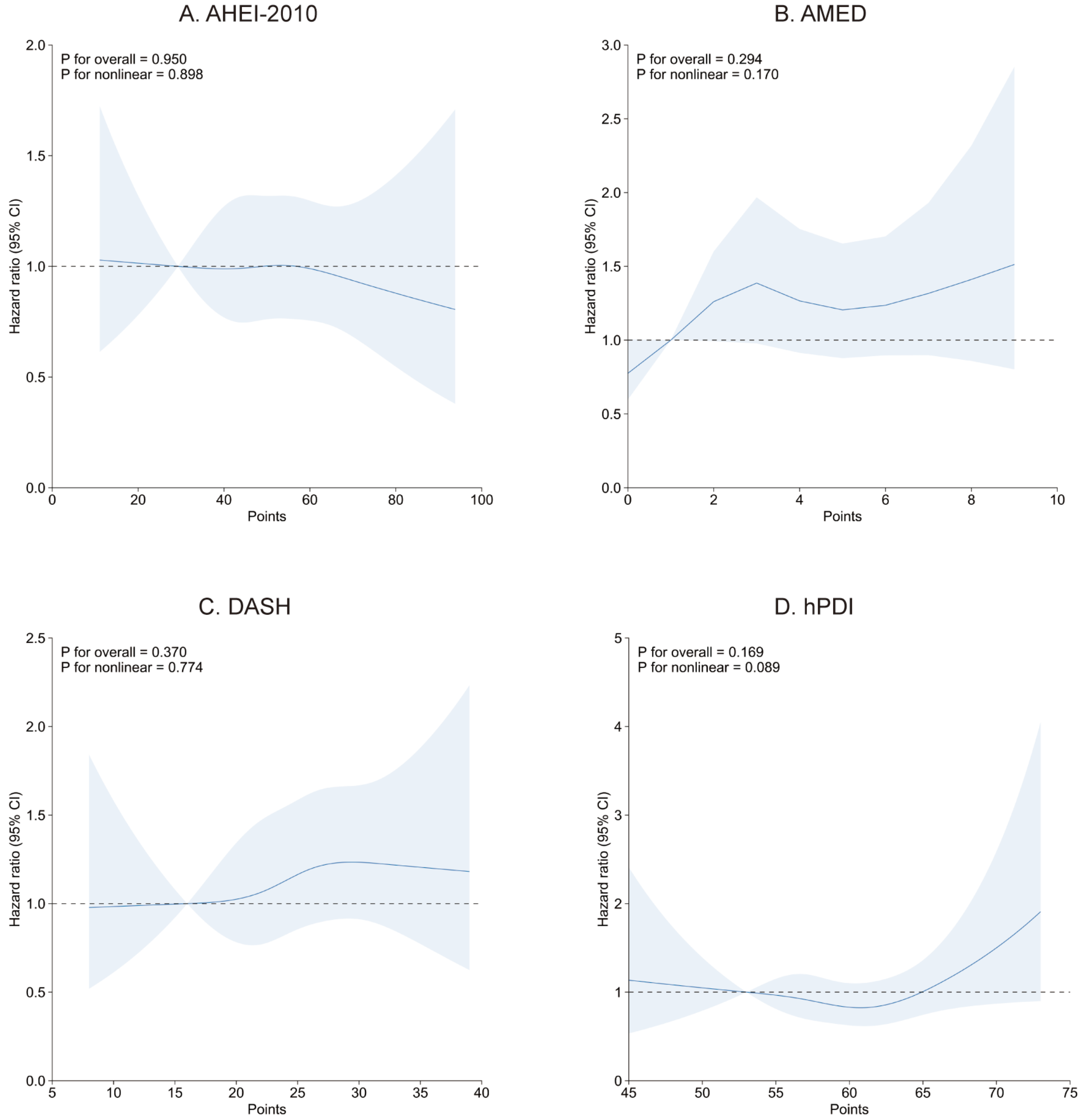

- **Supplementary Figure 4.** Hazard ratios (95% confidence interval) of vascular dementia according to diet quality scores in the UK Biobank cohort

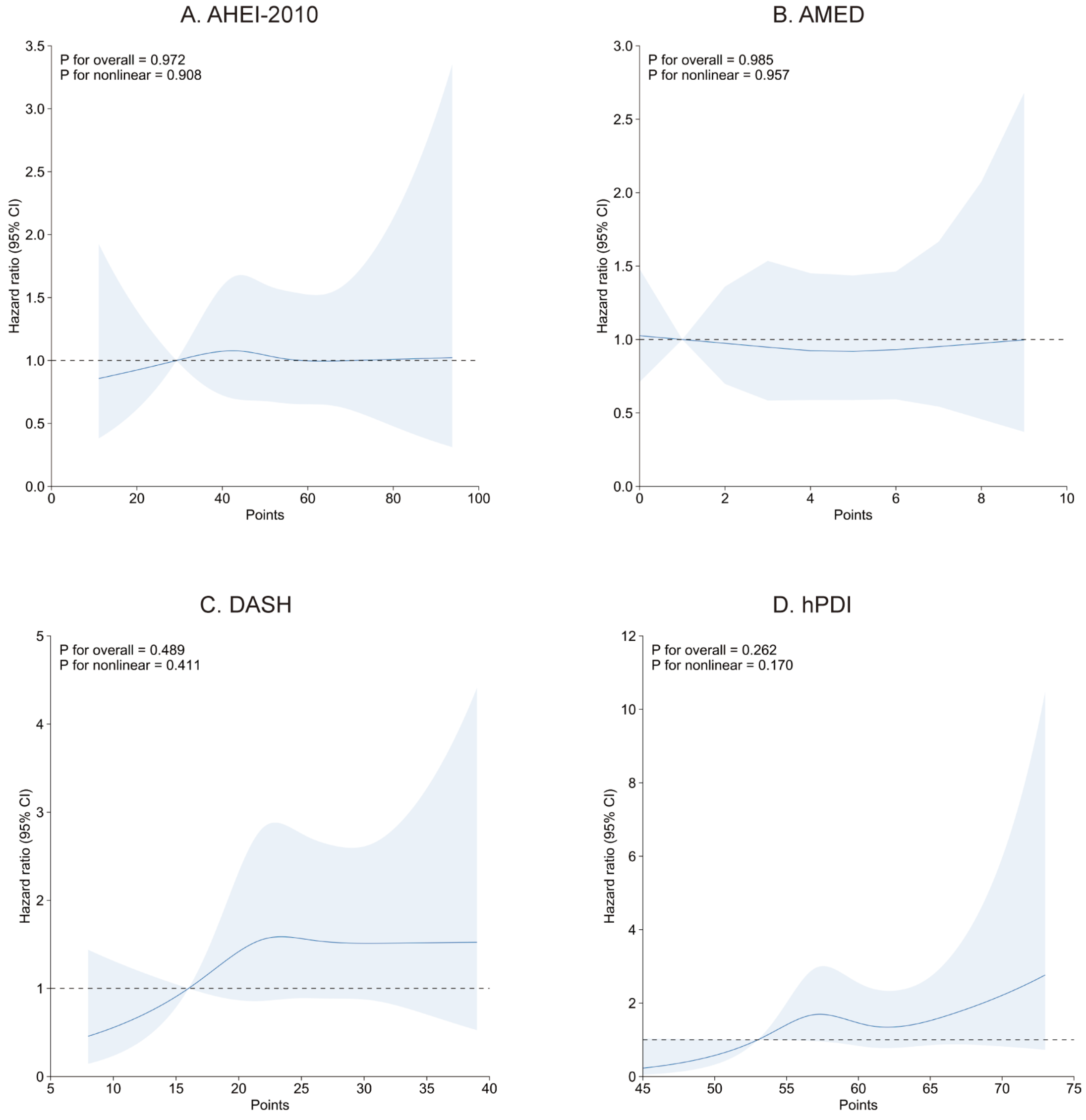

- **Supplementary Figure 5.** Hazard ratios (95% confidence interval) of depression according to diet quality scores in the UK Biobank cohort

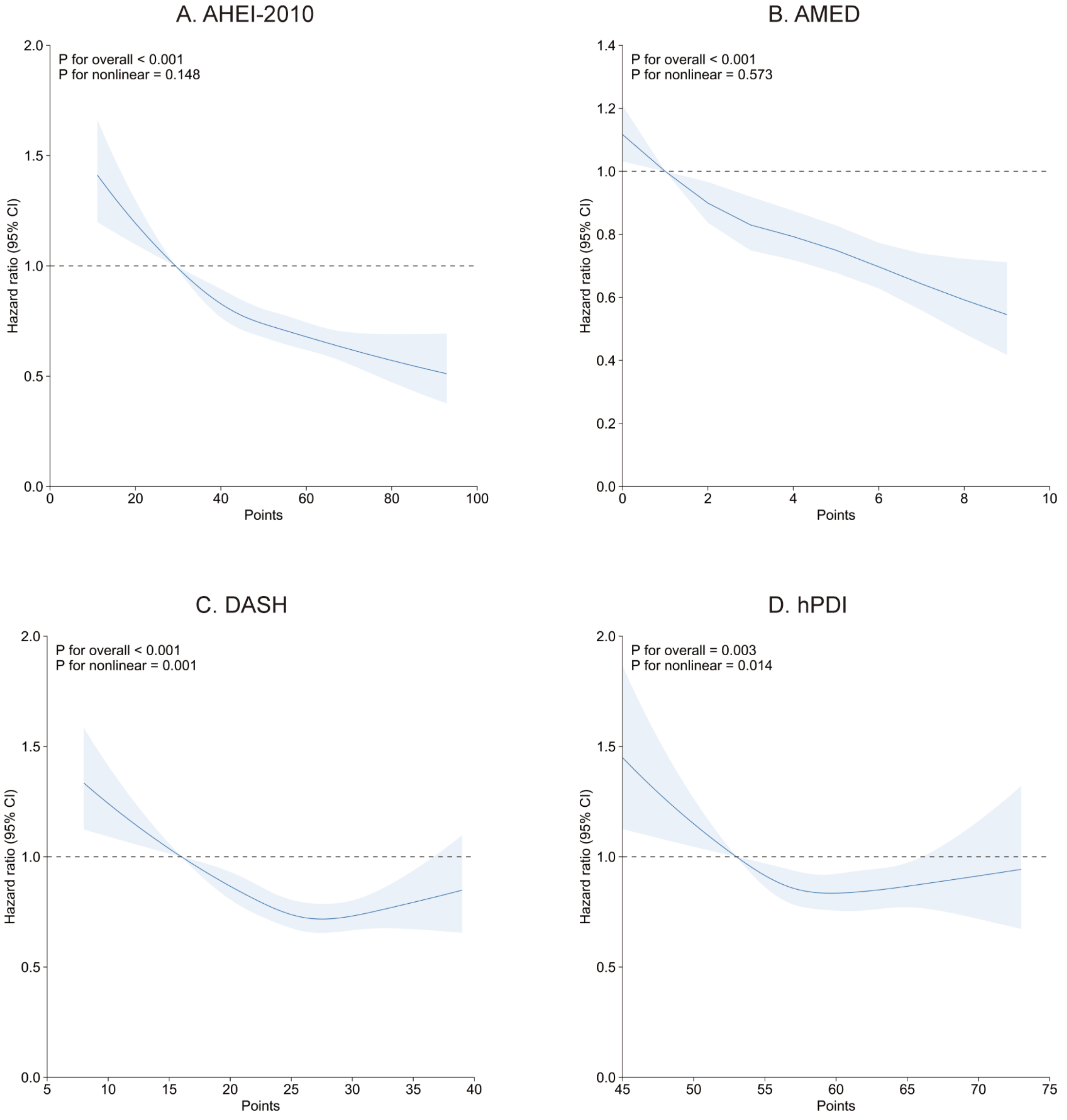

- **Supplementary Figure 6.** Hazard ratios (95% confidence interval) of phobic anxiety disorders according to diet quality scores in the UK Biobank cohort

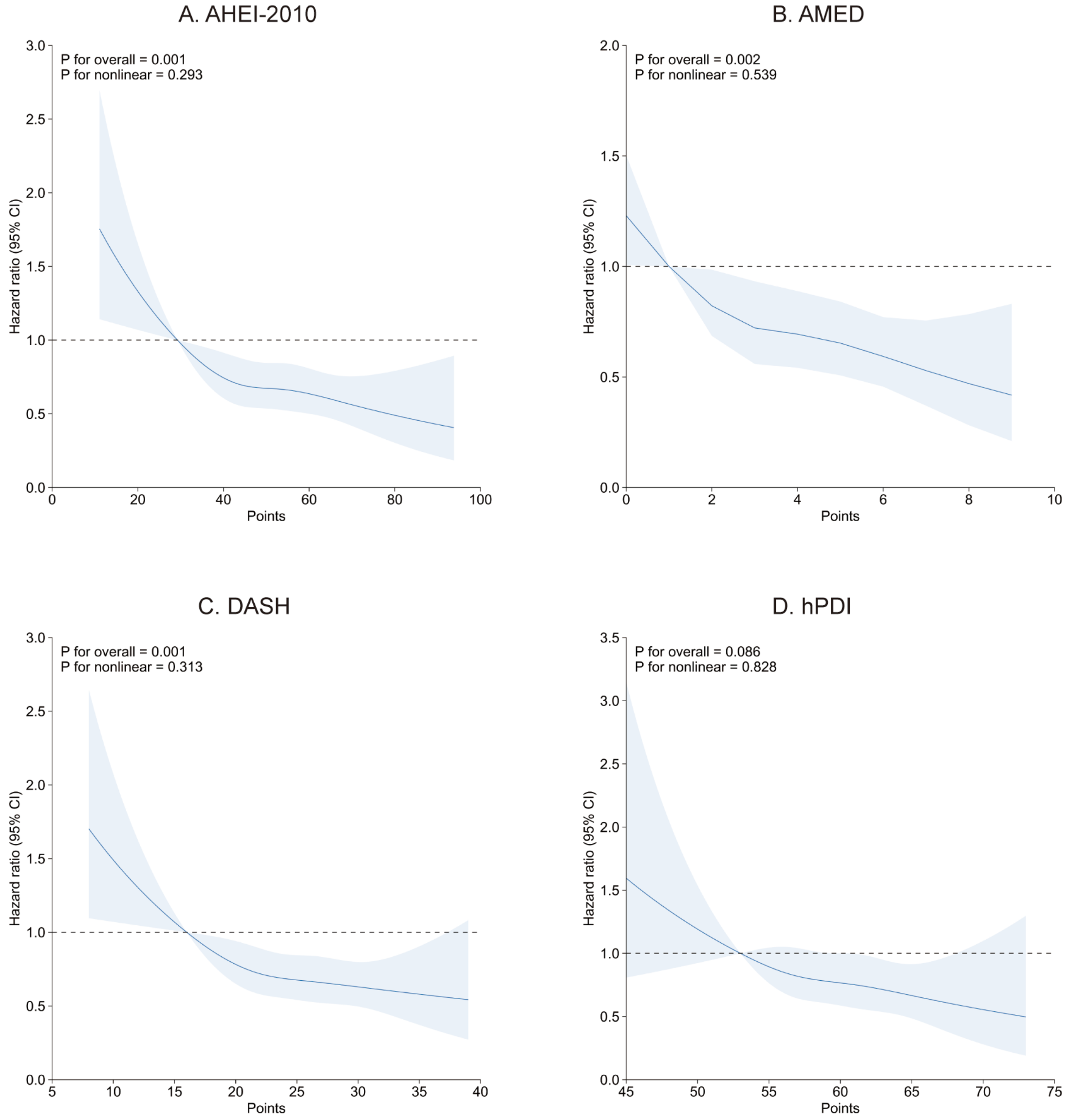

- **Supplementary Figure 7.** Hazard ratios (95% confidence interval) of other anxiety disorders according to diet quality scores in the UK Biobank cohort

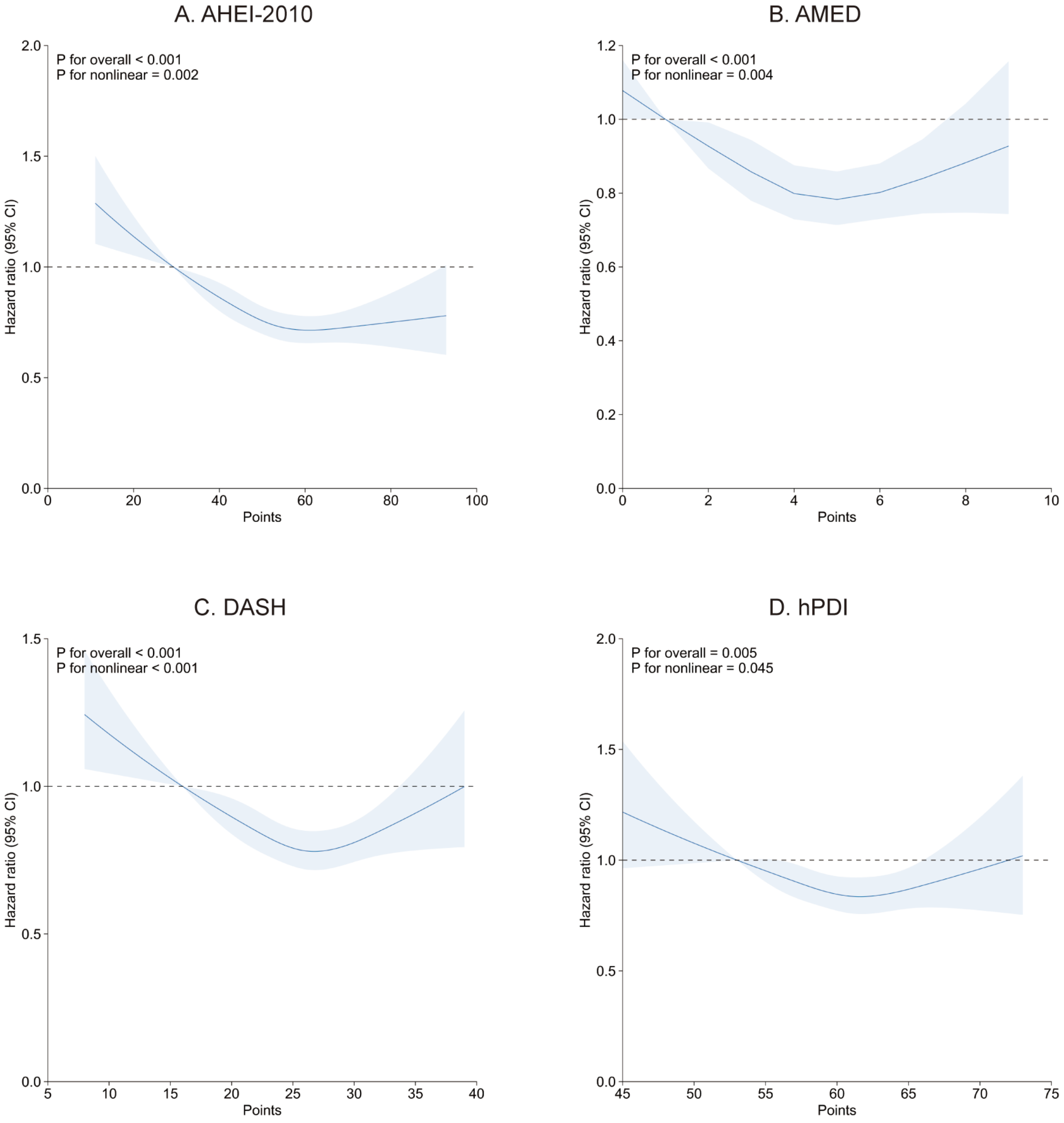

- **Supplementary Table 7.** Stratified analyses of the associations between four dietary patterns and risk of neuropsychiatric disorders, with P for interaction.

Subgroup	AMED		AHEI-2010		DASH		hPDI	
	HR (95%CI)	P for interaction	HR (95%CI)	P for interaction	HR (95%CI)	P for interaction	HR (95%CI)	P for interaction
Age<65	0.971 (0.934, 1.010)		0.956 (0.925, 0.988)		0.973 (0.942, 1.005)		0.984 (0.944, 1.025)	
Age⩾65	0.941 (0.875, 1.012)	0.742	0.923 (0.866, 0.984)	0.385	0.981 (0.921, 1.044)	0.394	0.997 (0.923, 1.077)	0.794
Female	0.939 (0.899, 0.981)		0.920 (0.886, 0.956)		0.954 (0.919, 0.990)		0.937 (0.894, 0.982)	
Male	0.985 (0.932, 1.042)	0.112	0.966 (0.921, 1.012)	0.062	0.973 (0.929, 1.019)	0.190	1.040 (0.982, 1.102)	0.007
BMI<25	0.976 (0.924, 1.031)		0.955 (0.909, 1.002)		0.966 (0.921, 1.013)		0.979 (0.922, 1.040)	
BMI⩾25	0.928 (0.888, 0.970)	0.218	0.911 (0.878, 0.945)	0.159	0.943 (0.909, 0.978)	0.346	0.966 (0.922, 1.011)	0.507
Never smoker	0.974 (0.928, 1.021)		0.938 (0.900, 0.978)		0.962 (0.923, 1.001)		0.974 (0.925, 1.024)	
Former smoker	0.950 (0.899, 1.003)		0.954 (0.910, 1.000)		0.975 (0.931, 1.020)		0.973 (0.918, 1.031)	
Current smoker	0.890 (0.789, 1.005)	0.439	0.876 (0.797, 0.964)	0.335	0.919 (0.834, 1.012)	0.432	1.024 (0.909, 1.154)	0.495
Sleep duration < 7	0.999 (0.933, 1.070)		0.931 (0.878, 0.986)		0.967 (0.913, 1.023)		1.028 (0.957, 1.105)	
Sleep duration ⩾7	0.943 (0.907, 0.982)	0.255	0.940 (0.909, 0.973)	0.715	0.961 (0.930, 0.994)	0.998	0.960 (0.921, 1.002)	0.189
Low PA	0.914 (0.861, 0.971)		0.903 (0.860, 0.950)		0.927 (0.882, 0.974)		0.978 (0.919, 1.041)	
Moderate PA	1.021 (0.961, 1.085)		0.968 (0.918, 1.021)		1.023 (0.972, 1.078)		1.035 (0.969, 1.104)	
High PA	0.950 (0.896, 1.007)	0.026	0.951 (0.904, 1.001)	0.032	0.950 (0.903, 0.998)	0.003	0.931 (0.874, 0.991)	0.033

- **Supplementary Table 8.** Association between AHEI-2010 score and ten metabolic biomarkers.

Metabolites	Beta	P value
Cholesterol/Total Lipids in Very Large VLDL	0.047	<0.001
Triglycerides/Total Lipids in Very Large VLDL	-0.047	<0.001
PUFA/TFA	0.044	<0.001
Triglycerides/Total Lipids in Medium VLDL	-0.041	<0.001
Cholesteryl Esters/Total Lipids in Very Large VLDL	0.036	<0.001
Cholesterol/Total Lipids in Medium VLDL	0.035	<0.001
Linoleic Acid/TFA	0.035	<0.001
Omega-6/TFA	0.031	<0.001
Cholesterol/Total Lipids in Large HDL	0.030	<0.001
Cholesterol/Total Lipids in Chylomicrons and Extremely Large VLDL	0.028	0.020

- **Supplementary Table 9.** Association between AMED score and ten metabolic biomarkers.

Metabolites	Beta	P value
PUFA/TFA	0.312	<0.001
Cholesterol/Total Lipids in Very Large VLDL	0.254	<0.001
Triglycerides/Total Lipids in Very Large VLDL	-0.252	<0.001
Omega-6/Omega-3 Ratio	-0.205	<0.001
SFA/TFA	-0.196	<0.001
Cholesteryl Esters/Total Lipids in Very Large VLDL	0.190	<0.001
Linoleic Acid/TFA	0.180	<0.001
Omega-6/TFA	0.173	<0.001
Triglycerides/Total Lipids in Medium VLDL	-0.145	0.009
Omega-3/TFA	0.139	<0.001

- **Supplementary Table 10.** Association between DASH score and ten metabolic biomarkers.

Metabolites	Beta	P value
PUFA/TFA	0.094	<0.001
Triglycerides/Total Lipids in Very Large VLDL	-0.088	<0.001
Cholesterol/Total Lipids in Very Large VLDL	0.080	<0.001
Phospholipids/Total Lipids in Very Large HDL	0.072	<0.001
Linoleic Acid/TFA	0.067	<0.001
Triglycerides/Total Lipids in Medium VLDL	-0.066	0.001
Omega-6/TFA	0.065	<0.001
Cholesterol/Total Lipids in Large HDL	0.061	<0.001
Cholesterol/Total Lipids in Medium VLDL	0.059	<0.001
Cholesteryl Esters/Total Lipids in Very Large VLDL	0.057	<0.001

- **Supplementary Table 11.** Association between hPDI score and ten metabolic biomarkers.

Metabolites	Beta	P value
Triglycerides/Total Lipids in Chylomicrons and Extremely Large VLDL	-0.154	0.001
Cholesterol/Total Lipids in Chylomicrons and Extremely Large VLDL	0.149	<0.001
Cholesterol/Total Lipids in Very Large VLDL	0.116	<0.001
Triglycerides/Total Lipids in Very Large VLDL	-0.113	<0.001
PUFA/TFA	0.108	<0.001
Cholesteryl Esters/Total Lipids in Very Large VLDL	0.090	<0.001
Omega-6/Omega-3 Ratio	-0.090	<0.001
Free Cholesterol/Total Lipids in Chylomicrons and Extremely Large VLDL	0.077	<0.001
Cholesteryl Esters/Total Lipids in Chylomicrons and Extremely Large VLDL	0.072	0.016
Triglycerides/Total Lipids in Medium VLDL	-0.069	0.012

- **Supplementary Figure 8.** Structural equation models examining the associations of dietary pattern scores with depression, mediated by inflammation and metabolic function.

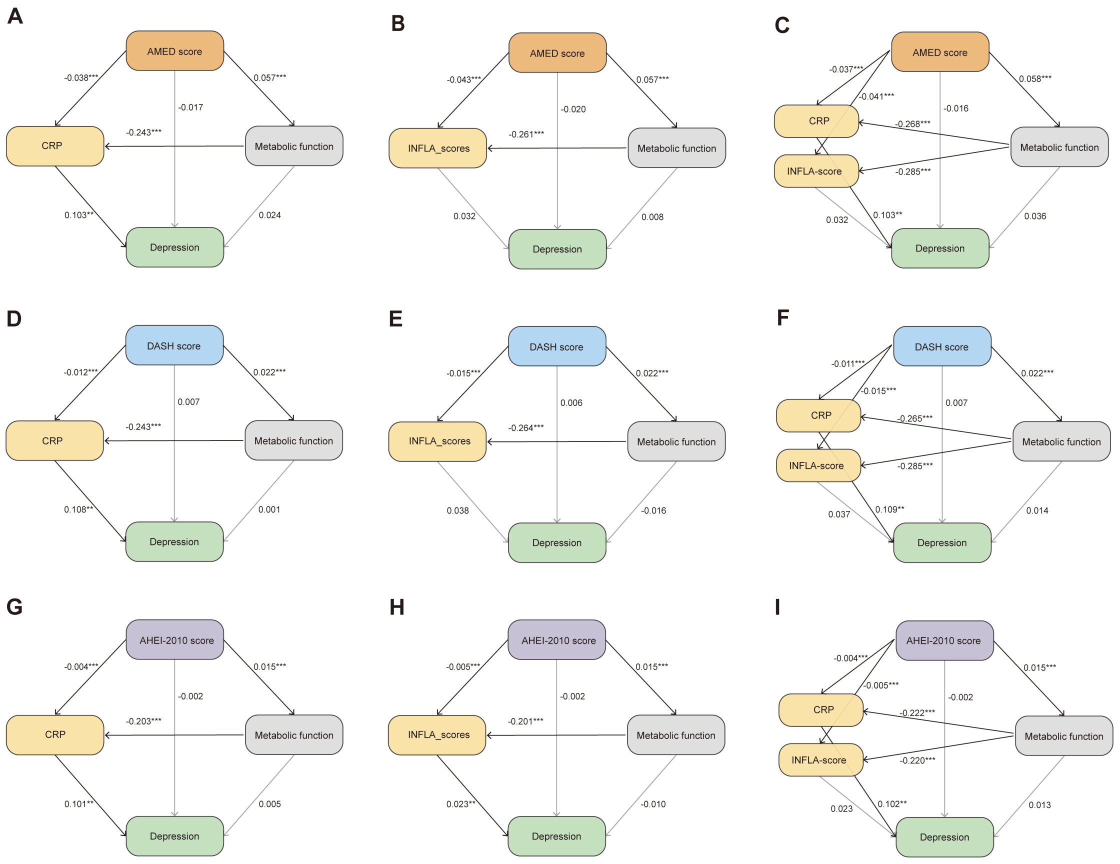

Supplementary Figure 8. Goodness-of-fit indices (CFI, TLI, RMSEA with 90% CI, and SRMR) for structural equation models. Panel A: CFI = 0.779; TLI = 0.765; RMSEA = 0.313 (90% CI: 0.310–0.316); SRMR = 0.207. Panel B: CFI = 0.779; TLI = 0.765; RMSEA = 0.313 (90% CI: 0.310–0.316); SRMR = 0.207. Panel C: CFI = 0.773; TLI = 0.754; RMSEA = 0.297 (90% CI: 0.294–0.299); SRMR = 0.198. Panel D: CFI = 0.957; TLI = 0.953; RMSEA = 0.144 (90% CI: 0.141–0.147); SRMR = 0.109. Panel E: CFI = 0.957; TLI = 0.954; RMSEA = 0.143 (90% CI: 0.140–0.146); SRMR = 0.108. Panel F: CFI = 0.949; TLI = 0.944; RMSEA = 0.146 (90% CI: 0.143–0.149); SRMR = 0.113. Panel G: CFI = 0.968; TLI = 0.965; RMSEA = 0.121 (90% CI: 0.118–0.124); SRMR = 0.099. Panel H: CFI = 0.779; TLI = 0.765; RMSEA = 0.313 (90% CI: 0.310–0.316); SRMR = 0.207. Panel I: CFI = 0.959; TLI = 0.954; RMSEA = 0.127 (90% CI: 0.124–0.130); SRMR = 0.105).

- **Supplementary Figure 9.** Structural equation models examining the associations between Alternative Healthy Eating Index-2010 dietary pattern and anxiety disorders, mediated by inflammation and metabolic function.

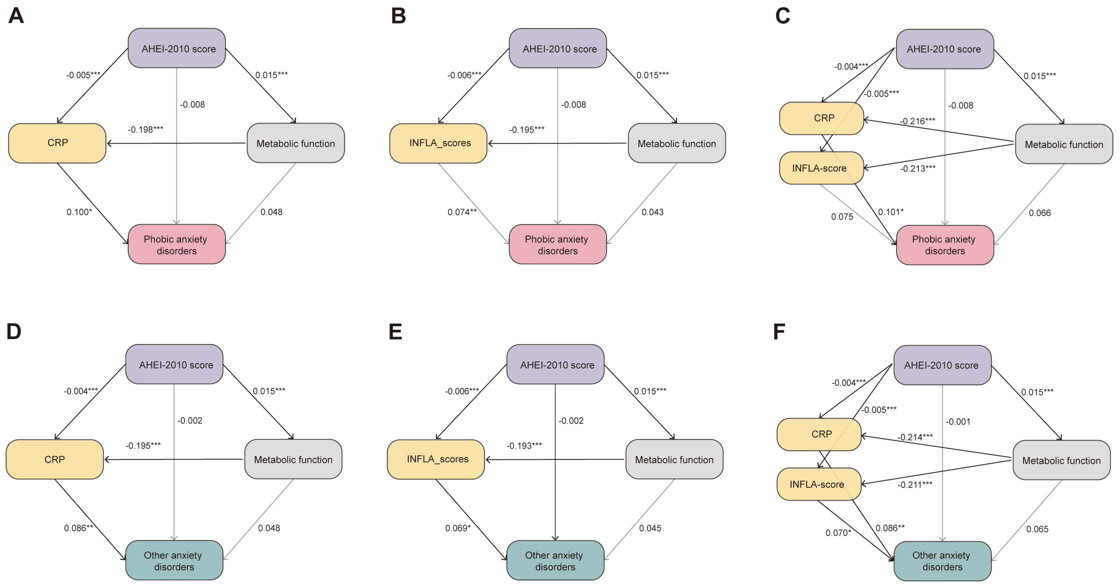

Supplementary Figure 9. Goodness-of-fit indices (CFI, TLI, RMSEA with 90% CI, and SRMR) for structural equation models. Panel A: CFI = 0.969; TLI = 0.966; RMSEA = 0.118 (90% CI: 0.115–0.121); SRMR = 0.097. Panel B: CFI = 0.969; TLI = 0.966; RMSEA = 0.117 (90% CI: 0.114–0.120); SRMR = 0.097. Panel C: CFI = 0.959; TLI = 0.955; RMSEA = 0.125 (90% CI: 0.122–0.128); SRMR = 0.105. Panel D: CFI = 0.969; TLI = 0.966; RMSEA = 0.117 (90% CI: 0.114–0.120); SRMR = 0.097. Panel E: CFI = 0.969; TLI = 0.967; RMSEA = 0.117 (90% CI: 0.114–0.120); SRMR = 0.097. Panel F: CFI = 0.959; TLI = 0.955; RMSEA = 0.125 (90% CI: 0.122–0.128); SRMR = 0.105).

- **Supplementary Figure 10.** Structural equation models examining the associations between healthy dietary pattern and neuropsychiatric disorders, mediated by a PCA-derived inflammation component and metabolic function.

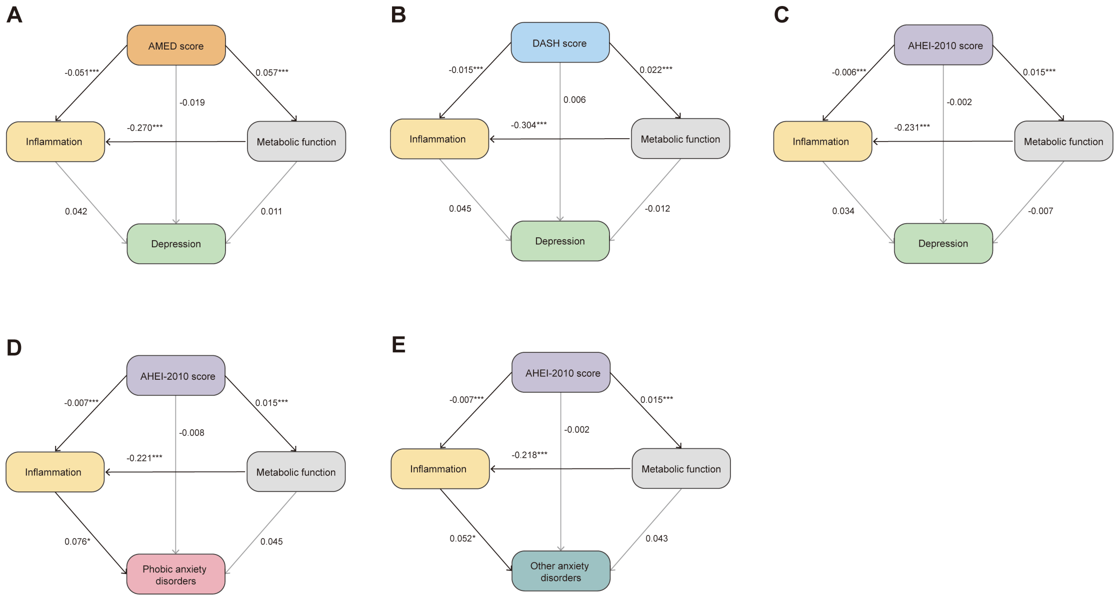

Supplementary Figure 10. Goodness-of-fit indices (CFI, TLI, RMSEA with 90% CI, and SRMR) for structural equation models. Panel A: CFI = 0.778; TLI = 0.764; RMSEA = 0.314 (90% CI: 0.311–0.316); SRMR = 0.206. Panel B: CFI = 0.957; TLI = 0.954; RMSEA = 0.143 (90% CI: 0.140–0.146); SRMR = 0.108. Panel C: CFI = 0.968; TLI = 0.966; RMSEA = 0.120 (90% CI: 0.117–0.123); SRMR = 0.098. Panel D: CFI = 0.969; TLI = 0.967; RMSEA = 0.117 (90% CI: 0.114–0.120); SRMR = 0.097. Panel E: CFI = 0.969; TLI = 0.967; RMSEA = 0.116 (90% CI: 0.113–0.119); SRMR = 0.097).

- **Supplementary Table 12.** Hazard ratios (95% confidence intervals) for all-cause dementia according to tertiles or per 20-percentile increase in adherence to diet quality scores in the UK Biobank cohort, after excluding participants who developed dementia within the first 2 years of follow-up.

Dietary pattern quality	Tertile1	Tertile2	Tertile3	Per 20-percentiles	p-trend
AMED	Ref.	0.888 (0.781, 1.009)	0.886 (0.752, 1.043)	0.979 (0.939, 1.021)	0.079
AHEI-2010	Ref.	0.939 (0.821, 1.075)	0.898 (0.782, 1.031)	0.969 (0.931, 1.008)	0.128
DASH	Ref.	0.941 (0.821, 1.078)	0.926 (0.809, 1.060)	0.992 (0.953, 1.032)	0.270
hPDI	Ref.	1.000 (0.857, 1.167)	0.979 (0.827, 1.159)	0.984 (0.939, 1.031)	0.805

- **Supplementary Table 13.** Hazard ratios (95% confidence intervals) for Alzheimer's disease according to tertiles or per 20-percentile increase in adherence to diet quality scores in the UK Biobank cohort, after excluding participants who developed Alzheimer's disease within the first 2 years of follow-up.

Dietary pattern quality	Tertile1	Tertile2	Tertile3	Per 20-percentiles	p-trend
AMED	Ref.	0.950 (0.782, 1.155)	1.043 (0.824, 1.322)	1.015 (0.952, 1.081)	0.850
AHEI-2010	Ref.	1.031 (0.837, 1.268)	0.964 (0.780, 1.190)	0.992 (0.934, 1.052)	0.700
DASH	Ref.	0.971 (0.783, 1.205)	1.151 (0.939, 1.411)	1.056 (0.993, 1.122)	0.144
hPDI	Ref.	1.018 (0.802, 1.292)	1.097 (0.851, 1.413)	0.997 (0.929, 1.071)	0.472

- **Supplementary Table 14.** Hazard ratios (95% confidence intervals) for Vascular dementia according to tertiles or per 20-percentile increase in adherence to diet quality scores in the UK Biobank cohort, after excluding participants who developed Vascular dementia within the first 2 years of follow-up.

Dietary pattern quality	Tertile1	Tertile2	Tertile3	Per 20-percentiles	p-trend
AMED	Ref.	0.899 (0.656, 1.231)	1.149 (0.789, 1.675)	1.031 (0.931, 1.142)	0.655
AHEI-2010	Ref.	0.818 (0.589, 1.136)	0.991 (0.719, 1.367)	1.019 (0.927, 1.120)	0.980
DASH	Ref.	1.255 (0.896, 1.758)	1.250 (0.895, 1.746)	1.090 (0.990, 1.201)	0.211
hPDI	Ref.	1.182 (0.808, 1.731)	1.212 (0.801, 1.835)	1.055 (0.940, 1.183)	0.364

- **Supplementary Table 15.** Hazard ratios (95% confidence intervals) for depression according to tertiles or per 20-percentile increase in adherence to diet quality scores in the UK Biobank cohort, after excluding participants who developed depression within the first 2 years of follow-up.

Dietary pattern quality	Tertile1	Tertile2	Tertile3	Per 20-percentiles	p-trend
AMED	Ref.	0.972 (0.897, 1.054)	0.886 (0.793, 0.989)	0.965 (0.940, 0.991)	0.042
AHEI-2010	Ref.	0.931 (0.856, 1.012)	0.885 (0.810, 0.965)	0.955 (0.932, 0.979)	0.006
DASH	Ref.	0.876 (0.805, 0.953)	0.930 (0.854, 1.013)	0.975 (0.951, 0.999)	0.063
hPDI	Ref.	0.933 (0.846, 1.028)	1.052 (0.945, 1.170)	1.006 (0.976, 1.036)	0.448

- **Supplementary Table 16.** Hazard ratios (95% confidence intervals) for phobic anxiety disorders according to tertiles or per 20-percentile increase in adherence to diet quality scores in the UK Biobank cohort, after excluding participants who developed phobic anxiety disorders within the first 2 years of follow-up.

Dietary pattern quality	Tertile1	Tertile2	Tertile3	Per 20-percentiles	p-trend
AMED	Ref.	0.902 (0.734, 1.109)	0.760 (0.571, 1.011)	0.930 (0.869, 0.995)	0.057
AHEI-2010	Ref.	0.897 (0.723, 1.113)	0.798 (0.637, 0.999)	0.945 (0.886, 1.007)	0.048
DASH	Ref.	0.861 (0.695, 1.068)	0.844 (0.678, 1.051)	0.941 (0.884, 1.003)	0.119
hPDI	Ref.	0.853 (0.662, 1.098)	0.793 (0.595, 1.056)	0.941 (0.870, 1.017)	0.104

- **Supplementary Table 17.** Hazard ratios (95% confidence intervals) for other anxiety disorders according to tertiles or per 20-percentile increase in adherence to diet quality scores in the UK Biobank cohort, after excluding participants who developed other anxiety disorders within the first 2 years of follow-up.

Dietary pattern quality	Tertile1	Tertile2	Tertile3	Per 20-percentiles	p-trend
AMED	Ref.	0.875 (0.813, 0.942)	0.939 (0.854, 1.033)	0.960 (0.938, 0.983)	0.028
AHEI-2010	Ref.	0.880 (0.815, 0.949)	0.855 (0.790, 0.924)	0.951 (0.930, 0.972)	<0.001
DASH	Ref.	0.875 (0.811, 0.944)	0.894 (0.828, 0.966)	0.966 (0.945, 0.987)	0.003
hPDI	Ref.	0.896 (0.822, 0.977)	0.916 (0.831, 1.009)	0.975 (0.949, 1.001)	0.053

- **Supplementary Figure 11.** The mediating role of inflammation in the associations between four healthy dietary patterns and major neuropsychiatric disorders.

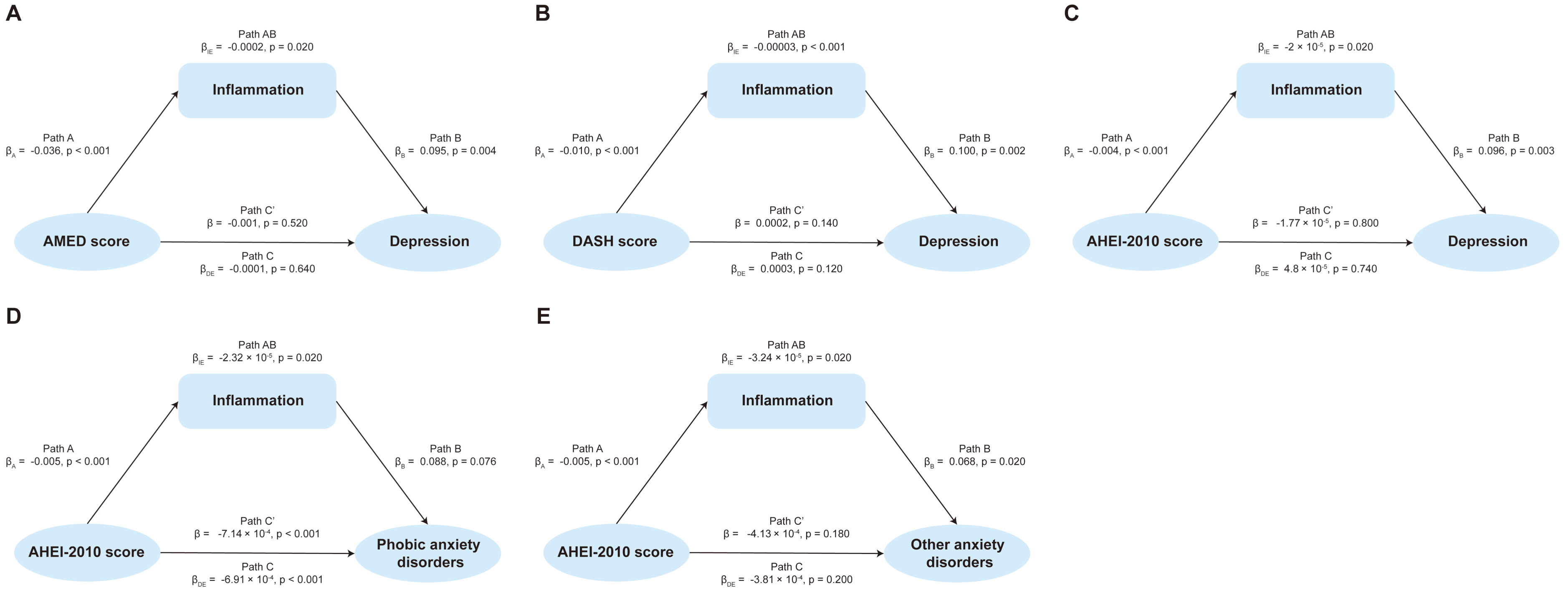

Supplementary Figure 11. In analysis A the mediation effect was not significant with a proportion of 10.8%. In analysis B the mediation was not significant with a proportion of 6.2%. In analysis C the mediation was not significant with a proportion of 4.7%. In analysis D the mediation was significant with a proportion of 3.6%. In analysis E the mediation was not significant with a proportion of 5.9%.

- **Supplementary Table 18.** Components and Scoring Criteria of Dietary Patterns

Component	AHEI-2010	AMED	DASH	hPDI
Fruit	[orange]	[orange]	[orange]	[orange]
Vegetables	[orange]	[orange]	[orange]	[orange]
Potatoes				[blue]
Whole grains	[orange]	[orange]	[orange]	[orange]
Refined grains				[blue]
Nuts	[orange]	[orange]	[orange]	[orange]
Legumes	[orange]	[orange]	[orange]	[orange]
Red meats and processed meats	[blue]	[blue]	[blue]	
Eggs				[blue]
Fish and seafood	[orange]	[orange]		[blue]
Meats				[blue]
Miscellaneous animal-based foods				[blue]
Sweets and desserts				[blue]
Animal fat				[blue]
Vegetable oils				
Sugar-sweetened beverages and fruits juices	[blue]			
Sugar-sweetened beverages			[blue]	[blue]
Fruit juices				[blue]
Total alcohol	[green]	[green]		
Tea and coffee				[orange]
Total dairy				[blue]
Low-fat dairy			[orange]	
Trans fat				
Long-chain omega-3 fatty acids				
Polyunsaturated fatty acids	[orange]			
Ratio of monounsaturated to saturated fatty acids		[orange]		
Ratio of polyunsaturated to saturated fatty acids				
Sodium	[blue]		[blue]	

[orange] Higher points or positive weights assigned to higher intakes of components.

[blue] Lower points or negative weights assigned to higher intakes of components

[green] Higher points assigned to moderate intakes of components

- **Supplementary Figure 12.** Conceptual framework of the study.

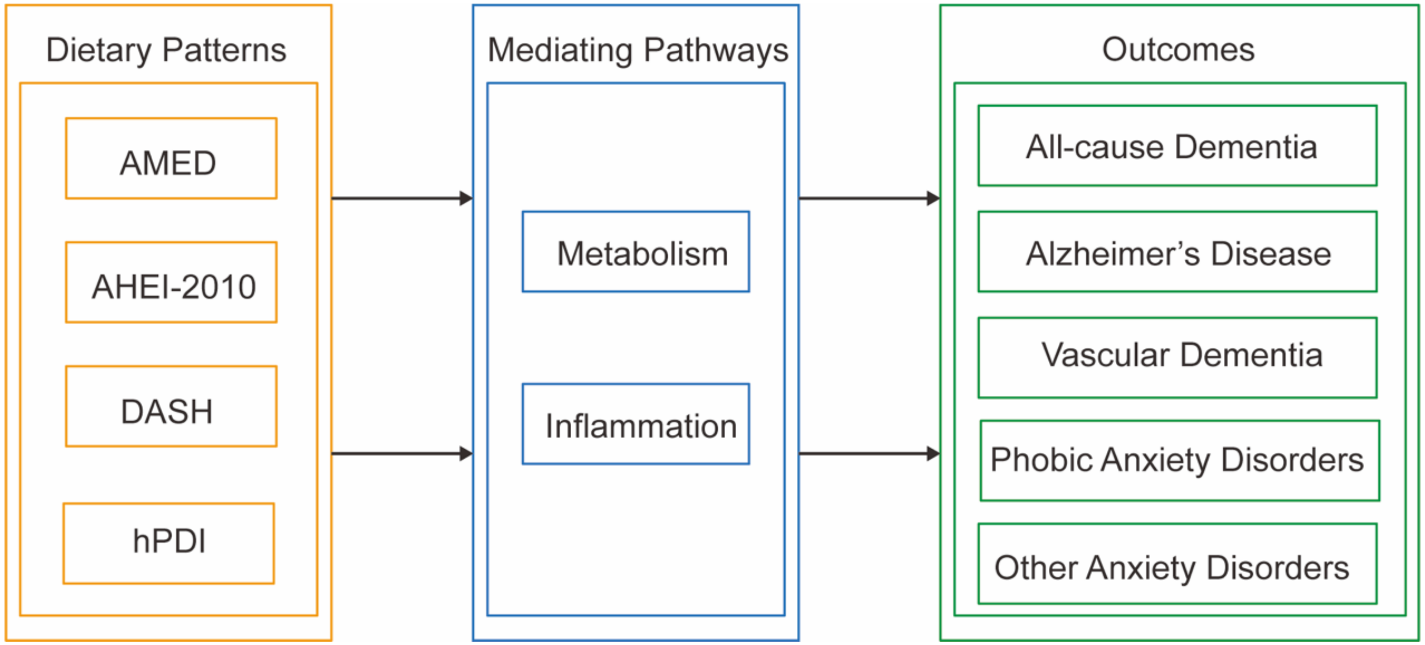

- **Supplementary Table 19.** Interaction effects between dietary pattern scores and metabolic markers on depression risk.

**Interaction term (Dietary pattern × Metabolite)**	HR (95% CI)	P for interaction
AMED * PUFA/TFA	1.011 (0.996, 1.025)	0.144
AMED * Cholesterol/Total Lipids in Very Large VLDL	1.014 (1.002, 1.026)	0.023
AMED * SFA/TFA	0.991 (0.978, 1.005)	0.216
DASH * PUFA/TFA	1.029 (0.998, 1.061)	0.064
DASH * Triglycerides/Total Lipids in Very Large VLDL	1.001 (0.967, 1.037)	0.955
DASH * Cholesterol/Total Lipids in Very Large VLDL	1.004 (0.974, 1.034)	0.806
AHEI-2010 * Triglycerides/Total Lipids in Very Large VLDL	0.997 (0.982, 1.012)	0.717
AHEI-2010 * Cholesterol/Total Lipids in Very Large VLDL	1.005 (0.992, 1.018)	0.491
AHEI-2010 * PUFA/TFA	1.007 (0.995, 1.020)	0.251

Cox proportional hazards models were fitted to examine the interaction effects between three dietary pattern scores (AMED, DASH, and AHEI-2010) and metabolic markers in relation to depression incidence. While ten metabolites were incorporated into the SEM framework, three representative metabolites most strongly associated with each dietary pattern were further selected based on regression results for interaction testing. Hazard ratios (HRs), 95% confidence intervals (CIs), and p-values are presented.

- **Supplementary Table 20.** Interaction effects between AHEI-2010 score and metabolic markers on phobic anxiety disorders risk.

**Interaction term (Dietary pattern × Metabolite)**	HR (95% CI)	P for interaction
AHEI-2010 * Triglycerides/Total Lipids in Very Large VLDL	0.984 (0.968, 1.000)	0.046
AHEI-2010 * Cholesterol/Total Lipids in Very Large VLDL	1.017 (1.001, 1.032)	0.035
AHEI-2010 * PUFA/TFA	1.010 (0.986, 1.035)	0.416

Cox proportional hazards models were fitted to examine the interaction effects between AHEI-2010 and metabolic markers in relation to phobic anxiety disorders incidence. While ten metabolites were incorporated into the SEM framework, three representative metabolites most strongly associated with each dietary pattern were further selected based on regression results for interaction testing. Hazard ratios (HRs), 95% confidence intervals (CIs), and p-values are presented.

- **Supplementary Table 21.** Interaction effects between AHEI-2010 score and metabolic markers on other anxiety disorders risk.

**Interaction term (Dietary pattern × Metabolite)**	HR (95% CI)	P for interaction
AHEI-2010 * Triglycerides/Total Lipids in Very Large VLDL	0.994 (0.982, 1.006)	0.285
AHEI-2010 * Cholesterol/Total Lipids in Very Large VLDL	1.007 (0.994, 1.019)	0.300
AHEI-2010 * PUFA/TFA	0.997 (0.986, 1.009)	0.616

Cox proportional hazards models were fitted to examine the interaction effects between AHEI-2010 and metabolic markers in relation to other anxiety disorders incidence. While ten metabolites were incorporated into the SEM framework, three representative metabolites most strongly associated with each dietary pattern were further selected based on regression results for interaction testing. Hazard ratios (HRs), 95% confidence intervals (CIs), and p-values are presented.

- **Supplementary Figure 13.** Mechanistic diagram illustrating the metabolic mediating pathways of dietary patterns through PUFA/TFA and VLDL composition on neuropathology.

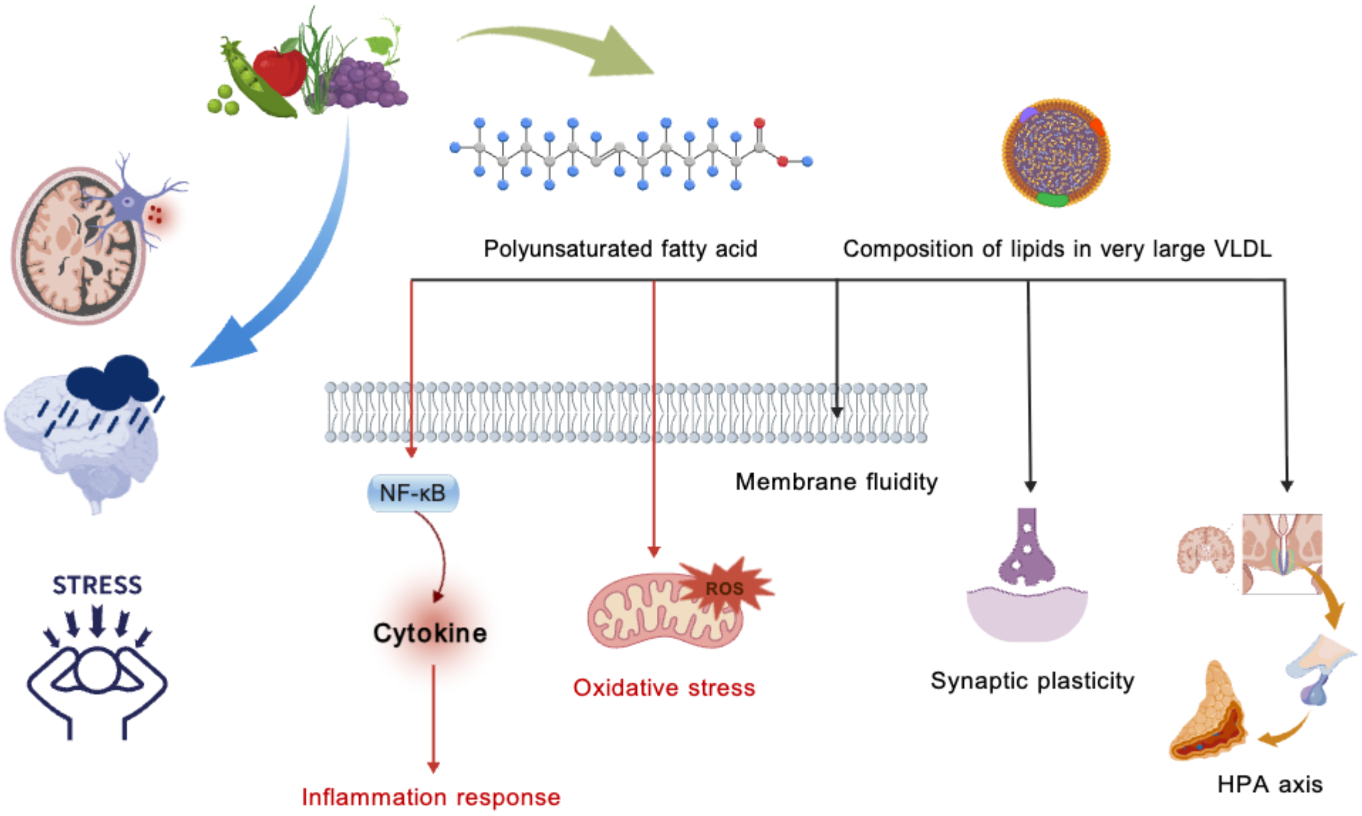

- **Supplementary Table 22.** Scoring for the Alternative Healthy Eating Index 2010 (AHEI-2010) in the UK Biobank.

Food groups	Food items	Criteria for 0 points	Criteria for 10 points
Vegetables (servings/d)	Broccoli, Cabbage, Cauliflower, Spinach, Sprouts, Garlic, Leek, Onion, Fresh tomato, Tinned tomato, Vegetarian sausages/burgers, Quorn, Other vegetarian alternative, Mixed vegetable, Vegetable pieces, Coleslaw, Side salad, Avocado, Beetroot, Butternut squash, Carrot, Celery, Courgette, Lettuce, Mushroom, Parsnip, Sweet pepper, Turnip/swede, Watercress, Other vegetables	0	≥5
Fruits (servings/d)	Stewed fruit, Prune, Dried fruit, Mixed fruit, Apple, Banana, Berry, Cherry, Grapefruit, Grape, Mango, Melon, Orange, Satsuma, Peach/nectarine, Pear, Pineapple, Plum, Other fruit	0	≥4
Whole grains (serving/d)	Porridge, Muesli, Oat crunch, Sweetened cereal, Plain cereal, Bran cereal, Whole-wheat cereal, Other cereal, Wholemeal pasta, Brown rice, Other grain intake	0	Women ≥5<br>Men ≥6
Nuts and legumes (serving/d)	Tofu, Baked bean, Pulses, Broad bean, Green bean, Pea, Sweetcorn, Salted peanuts, Unsalted peanuts, Salted nuts, Unsalted nuts, Seeds	0	≥1
Red/processed meat (servings/d)	Beef, Pork, Lamb, Sausage, Bacon, Ham	≥1.5	0
Sugar-sweetened beverages (servings/d)	Orange juice, Grapefruit juice, Pure fruit/vegetable juice, Fizzy drink, Squash, Fruit smoothie, Dairy smoothie, Hot chocolate, Low calorie drink, Low calorie hot chocolate	≥1	0
Trans fat (% of energy)	---------	≥4	≤0.5
Seafood (servings/d)	Sushi, Tinned tuna, Oily fish, White fish, Prawns, Lobster/crab, Shellfish, Other fish	0	≥2
PUFA (% of energy)	n-3 fatty acids, n-6 fatty acids	≤ 2	≥ 10
Sodium (mg/d)	Sodium	Highest decile	Lowest decile
Alcohol consumption (drinks/d)	Beer/cider, Spirits, Fortified wine, Red wine, White wine	Women ≥2.5<br>Men ≥3.5	0.5 – 1.5<br>0.5 – 2.0
Total		0	110

- **Supplementary Table 23.** Scoring for the Alternate Mediterranean Diet score (AMED) in the UK Biobank.

Food groups	Food items	Criteria for 0 points	Criteria for 1 points
Vegetables (servings/d)	Broccoli, Cabbage, Cauliflower, Spinach, Sprouts, Garlic, Leek, Onion, Fresh tomato, Tinned tomato, Vegetarian sausages/burgers, Quorn, Other vegetarian alternative, Mixed vegetable, Vegetable pieces, Coleslaw, Side salad, Avocado, Beetroot, Butternut squash, Carrot, Celery, Courgette, Lettuce, Mushroom, Parsnip, Sweet pepper, Turnip/swede, Watercress, Other vegetables	Below the median	Above the median
Fruits (servings/d)	Stewed fruit, Prune, Dried fruit, Mixed fruit, Apple, Banana, Berry, Cherry, Grapefruit, Grape, Mango, Melon, Orange, Satsuma, Peach/nectarine, Pear, Pineapple, Plum, Other fruit	Below the median	Above the median
Legumes (servings/d)	Tofu, Baked bean, Pulses, Broad bean, Green bean, Pea, Sweetcorn	Below the median	Above the median
Nuts (servings/d)	Salted peanuts, Unsalted peanuts, Salted nuts, Unsalted nuts, Seeds	Below the median	Above the median
Whole grains (serving/d)	Porridge, Muesli, Oat crunch, Sweetened cereal, Plain cereal, Bran cereal, Whole-wheat cereal, Other cereal, Wholemeal pasta, Brown rice, Other grain intake	Below the median	Above the median
Fish and seafood (servings/d)	Sushi, Tinned tuna, Oily fish, White fish, Prawns, Lobster/crab, Shellfish, Other fish	Below the median	Above the median
Red/processed meat (servings/d)	Beef, Pork, Lamb, Sausage, Bacon, Ham	Above the median	Below the median
Ratio of monounsaturated to saturated fat	Monounsaturated fatty acids, Saturated fatty acids	Below the median	Above the median
Alcohol (g/d)	Alcohol consumption	< 5 or > 15	5 – 15
Total		0	9

- **Supplementary Table 24.** Scoring for the Dietary Approaches to Stop Hypertension (DASH) in the UK Biobank.

Food groups	Food items	Score
Vegetables (servings/d)	Broccoli, Cabbage, Cauliflower, Spinach, Sprouts, Garlic, Leek, Onion, Fresh tomato, Tinned tomato, Vegetarian sausages/burgers, Quorn, Other vegetarian alternative, Mixed vegetable, Vegetable pieces, Coleslaw, Side salad, Avocado, Beetroot, Butternut squash, Carrot, Celery, Courgette, Lettuce, Mushroom, Parsnip, Sweet pepper, Turnip/swede, Watercress, Other vegetables	Positive
Fruits (servings/d)	Stewed fruit, Prune, Dried fruit, Mixed fruit, Apple, Banana, Berry, Cherry, Grapefruit, Grape, Mango, Melon, Orange, Satsuma, Peach/nectarine, Pear, Pineapple, Plum, Other fruit	Positive
Nuts and legumes (serving/d)	Tofu, Baked bean, Pulses, Broad bean, Green bean, Pea, Sweetcorn, Salted peanuts, Unsalted peanuts, Salted nuts, Unsalted nuts, Seeds	Positive
Whole grains (serving/d)	Porridge, Muesli, Oat crunch, Sweetened cereal, Plain cereal, Bran cereal, Whole-wheat cereal, Other cereal, Wholemeal pasta, Brown rice, Other grain intake	Positive
Low-fat dairy (servings/d)	Low fat yogurt, Rice/oat milk, Semi skimmed milk, Skimmed milk and cholesterol-lowering milk, Soy milk	Positive
Sodium (mg/d)	Sodium	Reverse
Red and processed meat (servings/d)	Beef, Pork, Lamb, Sausage, Bacon, Ham	Reverse
Sweetened beverages (servings/d)	Orange juice, Grapefruit juice, Pure fruit/vegetable juice, Fizzy drink, Squash, Fruit smoothie, Dairy smoothie, Hot chocolate, Low calorie drink, Low calorie hot chocolate	Reverse
Total		8 - 40

- **Supplementary Table 25.** Scoring for the healthful plant-based diet (hPDI) index in the UK Biobank.

Food groups	Food items	hPDI
Whole grains	Porridge, Muesli, Oat crunch, Sweetened cereal, Plain cereal, Bran cereal, Whole-wheat cereal, Other cereal, Wholemeal pasta, Brown rice, Other grain intake	Positive
Fruits	Stewed fruit, Prune, Dried fruit, Mixed fruit, Apple, Banana, Berry, Cherry, Grapefruit, Grape, Mango, Melon, Orange, Satsuma, Peach/nectarine, Pear, Pineapple, Plum, Other fruit	Positive
Vegetables	Broccoli, Cabbage, Cauliflower, Spinach, Sprouts, Garlic, Leek, Onion, Fresh tomato, Tinned tomato, Vegetarian sausages/burgers, Quorn, Other vegetarian alternative, Mixed vegetable, Vegetable pieces, Coleslaw, Side salad, Avocado, Beetroot, Butternut squash, Carrot, Celery, Courgette, Lettuce, Mushroom, Parsnip, Sweet pepper, Turnip/swede, Watercress, Other vegetables	Positive
Nuts	Salted peanuts, Unsalted peanuts, Salted nuts, Unsalted nuts, Seeds	Positive
Legumes	Tofu, Baked bean, Pulses, Broad bean, Green bean, Pea, Sweetcorn	Positive
Vegetable oils	Not available	---
Coffee & tea	Instant coffee, Filtered coffee, Cappuccino, Latte, Espresso, Other coffee type, Standard tea, Rooibos tea, Green tea, Herbal tea, Other tea	Positive
Fruit juices	Orange juice, Grapefruit juice, Pure fruit/vegetable juice	Reverse
Refined grains	Naan bread, Garlic bread, Crispbread, Oatcakes, Other bread, Double crust pastry, Single crust pastry, Crumble, Yorkshire pudding, Indian snacks, Chocolate-covered biscuits, Chocolate biscuits, Sweet biscuits, White pasta, White rice, Snackpot, Couscous	Reverse
Potatoes	Boiled/baked potatoes, Mashed potato, Sweet potato	Reverse
Sugar-sweetened beverages	Fizzy drink, Squash, Fruit smoothie, Dairy smoothie, Hot chocolate, Low calorie drink, Low calorie hot chocolate	Reverse
Sweets & desserts	Added sugars and preserves, Pancake, Scotch pancake, Croissant, Danish pastry, Scone, Ice-cream, Milk-based pudding, Other milk-based pudding, Soya dessert, Fruitcake, Cake, Doughnut, Sponge pudding, Cheesecake, Other dessert, Chocolate bar, White chocolate, Milk chocolate, Dark chocolate, Chocolate-covered raisin, Chocolate sweet, Diet sweets, Sweets, Cereal bar, Other sweets	Reverse
Animal fat	Animal fat spread (normal), Animal fat spread (lower fat)	Reverse
Dairy	Low fat yogurt, Rice/oat milk, Semi skimmed milk, Skimmed milk and cholesterol-lowering milk, Soy milk, Full fat yogurt, Whole milk, Cream, Low fat hard cheese, Hard cheese, Soft cheese, Blue cheese, Low fat cheese spread, Cheese spread, Cottage cheese, Feta, Mozzarella, Goat's cheese, Other cheese	Reverse
Egg	Whole egg, Omelette, Eggs in sandwiches, Scotch egg, Other egg	Reverse
Seafood	Sushi, Tinned tuna, Oily fish, White fish, Prawns, Lobster/crab, Shellfish, Other fish	Reverse
Meat	Beef, Pork, Lamb, Sausage, Bacon, Ham, Poultry, Liver, Other meat	Reverse
Miscellaneous animal-based foods	Pizza, Indian snacks	Reverse

- **Supplementary Table 26.** ICD-10 codes used for incident of the major neuropsychiatric disease.

Diagnostic category	Specific diagnosis	Field code
Dementia	Dementia in Alzheimer's disease	F00
	Vascular dementia	F01
	Unspecified dementia	F03
Depression	Depressive episode	F32
Anxiety	Phobic anxiety disorders	F40
	Other anxiety disorders	F41
